# Trimethoprim-Sulfamethoxazole and Acute Respiratory Failure in Adolescents and Young Adults

**DOI:** 10.1001/jamanetworkopen.2025.45251

**Published:** 2025-11-24

**Authors:** Fatemeh Ahmadi, Eric McArthur, Facundo Garcia-Bournissen, Michael J. Rieder, Flory T. Muanda

**Affiliations:** 1 ICES, Toronto, Ontario, Canada; 2 Department of Epidemiology and Biostatistics, Western University, London, Ontario, Canada; 3 London Health Sciences Centre Research Institute, London Health Sciences Centre, London, Ontario, Canada; 4 Department of Pediatrics, Western University, London, Ontario, Canada; 5 Department of Physiology and Pharmacology, Western University, London, Ontario, Canada

## Abstract

**Importance:**

The US Food and Drug Administration (FDA) has issued a warning and a label change regarding a potential association between trimethoprim-sulfamethoxazole (TMP-SMX) and acute respiratory failure in healthy adolescents and young adults.

**Objective:**

To examine the 30-day risk of a hospital visit (ie, hospitalization or emergency department visit) with acute respiratory failure in adolescents and young adults aged 10 to younger than 25 years old newly dispensed oral TMP-SMX compared with new users of amoxicillin or a cephalosporin.

**Design, Setting, and Participants:**

This retrospective, population-based, new-user cohort study in Ontario, Canada (2003-2023) used linked administrative health care data. The TMP-SMX vs amoxicillin and TMP-SMX vs cephalosporins cohorts both included adolescents and young adults aged 10 to younger than 25 years who were newly dispensed oral TMP-SMX, amoxicillin, or cephalosporins for 3 or more days from an outpatient pharmacy. Data were analyzed from January 1 to April 30, 2025.

**Exposure:**

TMP-SMX for 3 days or more.

**Main Outcomes and Measures:**

The primary outcome was a composite outcome of the 30-day risk of a hospital visit with acute respiratory failure (defined as a diagnosis of acute respiratory failure or receipt of mechanical ventilation, tracheotomy, or extracorporeal membrane oxygenation). Secondary outcomes were the individual components of the composite outcome, all-cause hospitalization, and all-cause mortality. Overlap weighting on the propensity score was used to balance comparison groups on 84 indicators of baseline health. Weighted risk ratios were obtained using log-binomial regression and weighted risk differences using binomial regression. Sensitivity analyses using a negative control outcome, and case-crossover analysis were also performed.

**Results:**

The TMP-SMX vs amoxicillin cohort included 575 218 individuals (44 801 TMP-SMX users and 530 417 amoxicillin users; median age after weighting, 19 years [IQR, 16-22 years]; 74.3% female). The TMP-SMX vs cephalosporins cohort included 248 236 individuals (51 197 TMP-SMX users and 197 039 cephalosporin users; median age after weighting, 19 years [IQR, 16-22 years]; 72.3% were female). The risk of the composite outcome occurred in 15 of 44 801 patients (0.03%) who started TMP-SMX and in 49 of 530 417 (0.01%) who started amoxicillin (number of weighted events, 7 of 21 579 [0.03%] for TMP-SMX and 2 of 21 579 [0.01%] for amoxicillin; weighted risk ratio, 2.79 [95% CI, 1.01-7.71]; weighted risk difference, 0.02% [95% CI, 0.001%-0.04%]). The risk of the composite outcome occurred in 17 of 51 197 patients (0.03%) who started TMP-SMX and in 21 of 197 039 (0.01%) who started cephalosporins (number of weighted events, 8 of 20 538 [0.04%] for TMP-SMX and 3 of 20 538 [0.01%] for cephalosporins; weighted risk ratio, 2.85 [95% CI, 1.11-7.31]; weighted risk difference, 0.02% [95% CI, 0.005%-0.05%]). Results were consistent in sensitivity analyses.

**Conclusions and Relevance:**

These findings suggest that the 30-day risk of a hospital visit with acute respiratory failure was higher among those receiving TMP-SMX compared with those receiving amoxicillin or cephalosporins. These findings supported the FDA warning, and if replicated, the risks should be carefully weighed against the benefits of TMP-SMX use. Regulatory agencies could reinforce the FDA warning, and product monographs and prescribing guidelines should be updated and revised accordingly.

## Key Points

**Question:**

Is the use of trimethoprim-sulfamethoxazole (TMP-SMX) associated with an increased 30-day risk of hospital visits for acute respiratory failure among healthy adolescents and young adults compared with amoxicillin or cephalosporins?

**Findings:**

In this cohort study of adolescents and young adults aged 10 to younger than 25 years in Ontario, Canada, initiation of TMP-SMX was associated with a significantly higher 30-day risk of a hospital visit for acute respiratory failure compared with amoxicillin and cephalosporins. The absolute risk increase was small (0.02%) but consistent across sensitivity analyses.

**Meaning:**

These findings support the US Food and Drug Administration’s warning regarding the risk of acute respiratory failure with TMP-SMX in healthy adolescents and young adults, and clinicians and regulators should carefully weigh this risk when prescribing TMP-SMX and consider updates to prescribing guidelines and product labeling.

## Teaser

This cohort study examines the 30-day risk of a hospital visit for acute respiratory failure among adolescents and young adults newly dispensed oral trimethoprim-sulfamethoxazole compared with new users of amoxicillin or a cephalosporin.

## Introduction

Trimethoprim-sulfamethoxazole (TMP-SMX) is a combination of 2 antibiotics primarily used to treat and prevent *Pneumocystis* pneumonia and serves as a first- or second-line treatment for various other infections, including urinary tract and skin infections.^1,2^ Severe cutaneous reactions—such as Stevens-Johnson syndrome, toxic epidermal necrolysis—and drug reaction with eosinophilia and systemic symptoms, as well as hyperkalemia and drug-induced liver injury, are known adverse events associated with TMP-SMX use. However, evidence linking TMP-SMX to acute respiratory failure is limited.

More than 19 case reports have associated acute respiratory failure with TMP-SMX use, particularly for acne treatment, in healthy adolescents and young adults.^3,4,5^ The median age in those studies was 16.5 years (range, 10-37 years). Symptoms of these conditions included severe shortness of breath, rapid breathing, hypoxemia (low blood oxygen levels), and, in severe cases, respiratory failure requiring mechanical ventilation.^5^ Although earlier literature documented instances of acute lung injury in adults, the severity of recent cases of acute respiratory failure—some resulting in lung transplant or death—prompted the US Food and Drug Administration (FDA) to issue a label change in 2019 about TMP-SMX use for healthy adolescents and young adults.^6,7^

To date, no population-based studies have been conducted, to our knowledge, to confirm this warning, and current evidence is primarily informed by case reports and case series involving individuals aged 10 to younger than 25 years (our search strategy is shown in eTable 1 and the results in eTable 2 in Supplement 1). To fill this gap and inform safe TMP-SMX prescribing, we conducted a population-based study to examine the 30-day risk of a hospital visit with acute respiratory failure in adolescents and young adults aged 10 to younger than 25 years who were new users of oral TMP-SMX compared with those who were new users of amoxicillin or a cephalosporin.

## Methods

### Study Design and Setting

This study was conducted using linked administrative health care databases in the province of Ontario, Canada (April 1, 2002, to August 1, 2023). All Ontario residents (approximately 15 million) have universal access to hospital care and physician services through a government-funded single-payer system. Individuals aged 24 years or younger without private insurance are eligible for provincial universal prescription drug coverage through the Ontario Health Insurance Plus.^8^ Additionally, those receiving financial assistance through Ontario Works or the Ontario Disability Support Program are eligible for prescription drug coverage under the Ontario Drug Benefit program.^9,10^ The use of data in this study was authorized under section 45 of Ontario’s Personal Health Information Protection Act, which does not require review by a research ethics board or informed consent.^11^ Study reporting followed the Strengthening the Reporting of Observational Studies in Epidemiology (STROBE) reporting guideline for observational studies that use routinely collected health data and the reporting of studies conducted using observational routinely collected health data statement for pharmacoepidemiology (RECORD-PE).^12,13^

### Data Sources

Patient characteristics, prescription drug use, covariate information, and outcome data were obtained from 8 health care databases at ICES (Institute for Clinical Evaluative Sciences).^14^ The datasets were linked using unique encoded identifiers and analyzed at ICES (Canadian Institute for Health Information–Discharge Abstract Database, ICES Physician Database, National Ambulatory Care Reporting System, Ontario Drug Benefit Database, Ontario Health Insurance Plan database, Ontario Laboratories Information System, Ontario Mental Health Reporting System, and the Registered Persons Database). Hospital admissions and diagnoses are coded by trained personnel using the *International Statistical Classification of Diseases and Related Health Problems, Tenth Revision* system; personnel only consider physician-recorded diagnoses in a patient’s medical record when assigning codes and do not review or interpret symptoms or test results. Additional information on the databases, variable definitions, and administrative codes is provided in eTable 3 in Supplement 1.

### Patients

Two separate cohorts were created to investigate the association between TMP-SMX use and the 30-day risk of a hospital visit with acute respiratory failure. The TMP-SMX vs amoxicillin cohort included adolescents and young adults aged 10 to younger than 25 years who were newly dispensed oral TMP-SMX or amoxicillin for 3 days or more from an outpatient pharmacy between April 1, 2002, and August 1, 2023. The TMP-SMX vs cephalosporins cohort included adolescents and young adults aged 10 to younger than 25 years who were newly dispensed oral TMP-SMX or a cephalosporin for 3 days or more from an outpatient pharmacy between April 1, 2002, and August 1, 2023. The prescription fill date was the individual’s cohort entry date (the index date); patients could only enter the cohort once. A diagram detailing the study design is provided in eFigure 1 in Supplement 1.^15^

To ensure that individuals were new users of the study antibiotics (ie, TMP-SMX, amoxicillin, or cephalosporins), those with any evidence of study antibiotics use (including combination drug prescriptions) in the 180 days before the index date were excluded. For each analysis, a distinct TMP-SMX cohort was constructed by excluding patients with prior use of either the study drug or the specific comparator within the 180 days before cohort entry. These analysis-specific exclusions result in different cohort sizes for TMP-SMX across comparisons. Those who were discharged from the hospital or emergency department within 2 days before the index date were also excluded (in Ontario, an individual who starts an antibiotic prescription during a hospital admission would have their outpatient prescription dispensed on the same day or the day after hospital discharge). Additional exclusion criteria are provided in eTable 4 in Supplement 1.

### Exposure

The primary exposure of interest was oral TMP-SMX. Given that the drug monographs recommend 3 days as the minimum duration of treatment with TMP-SMX, we restricted the cohort to those who were dispensed the medication for at least 3 days. Two active comparator groups, amoxicillin and cephalosporins, were chosen for the comparison to reduce the influence of indication bias.^16^ These medications were chosen based on their similarity with TMP-SMX in bacterial coverage and indications.^17,18,19,20^

### Outcomes

All primary and secondary outcomes were prespecified. The primary outcome was a composite outcome of the 30-day risk of a hospital visit with acute respiratory failure (defined as a diagnosis of acute respiratory failure or receipt of mechanical ventilation, tracheotomy, or extracorporeal membrane oxygenation [ECMO]). We chose this composite outcome because the inclusion of acute respiratory failure, mechanical ventilation, tracheotomy, and ECMO provides a more comprehensive measure of severe respiratory failure. These interrelated events include both diagnosis and critical management approaches for severe acute respiratory failure, capturing the full spectrum of its most serious manifestations. The outcome and time frame were defined based on a review of the literature (studies summarized in eTable 2 in Supplement 1); in these studies, the median time to acute respiratory failure after TMP-SMX initiation was 20.5 days (IQR, 16.2-24.0 days]). Secondary outcomes were the components of the composite outcome, 30-day all-cause hospitalization, and 30-day all-cause mortality. Diagnostic codes for all outcome variables and information on their interpretation are provided in eTable 5 in Supplement 1.

### Statistical Analysis

Analyses were conducted using SAS Enterprise Guide, version 8.3 (SAS Institute Inc). Overlap weighting was used to balance comparison groups on indicators of baseline health. The propensity score was estimated using multivariable logistic regression with 84 covariates chosen a priori (defined in eTable 6 in Supplement 1). These covariates were assessed within prespecified windows: comorbidities during the 5 years prior to the index date, medication use during the 120 days prior, health care use and investigations during the 1 year prior, and recent infections, health care use, and investigations within 7 days prior to or on the index date. Covariates were selected based on their potential to act as confounders, known risk factors for the outcomes, or proxies for such risk factors, guided by prior literature and clinical relevance.^21^ The overlap-weighting method (described in eTable 7 in Supplement 1) produces a weighted population with reasonable clinical equipoise for treatment decisions.^22,23^ Between-group differences in baseline characteristics were compared using standardized differences in both the unweighted and weighted samples^24^ (differences >10% were considered meaningful). Weighted risk ratios with 95% CIs were obtained using log-binomial regression,^25^ and weighted risk differences with 95% CIs were obtained using binomial regression with an identity link function. All variables in this study were complete except for the study antibiotics prescriber specialty (40% missing in the TMP-SMX vs amoxicillin cohort and 11% in the TMP-SMX vs cephalosporins cohort; defined as a separate category), patient income quintile (0.4% missing; recoded as the middle quintile), and urban or rural location of residence (0.3% missing; recoded as urban location of residence). Emigration from the province, which occurs at a rate of 0.5% per year, was the only potential reason for lost follow-up.^26^

Two post hoc sensitivity analyses were conducted to assess the robustness of the main results: (1) an analysis using a negative control outcome^27^ (defined as receipt of a nuclear medicine procedure, capturing patients with complex health profiles and high health care use, who are susceptible to similar confounders as the primary outcome) and (2) a case-crossover analysis.^28,29^ The details of the case-crossover design are provided in eTable 7 in Supplement 1. To estimate the association using this design, we calculated the odds ratio by comparing TMP-SMX exposure between the case and control periods, using discordant pairs—instances where individuals were exposed in the case period but not in the control period or vice versa. The odds ratio was calculated as the ratio of these discordant pairs, and *P* values were 2 sided. Data were analyzed from January 1 to April 30, 2025. *P* < .05 was interpreted as statistically significant.

## Results

### Patients

#### TMP-SMX vs Amoxicillin Cohort

**Figure 1.**
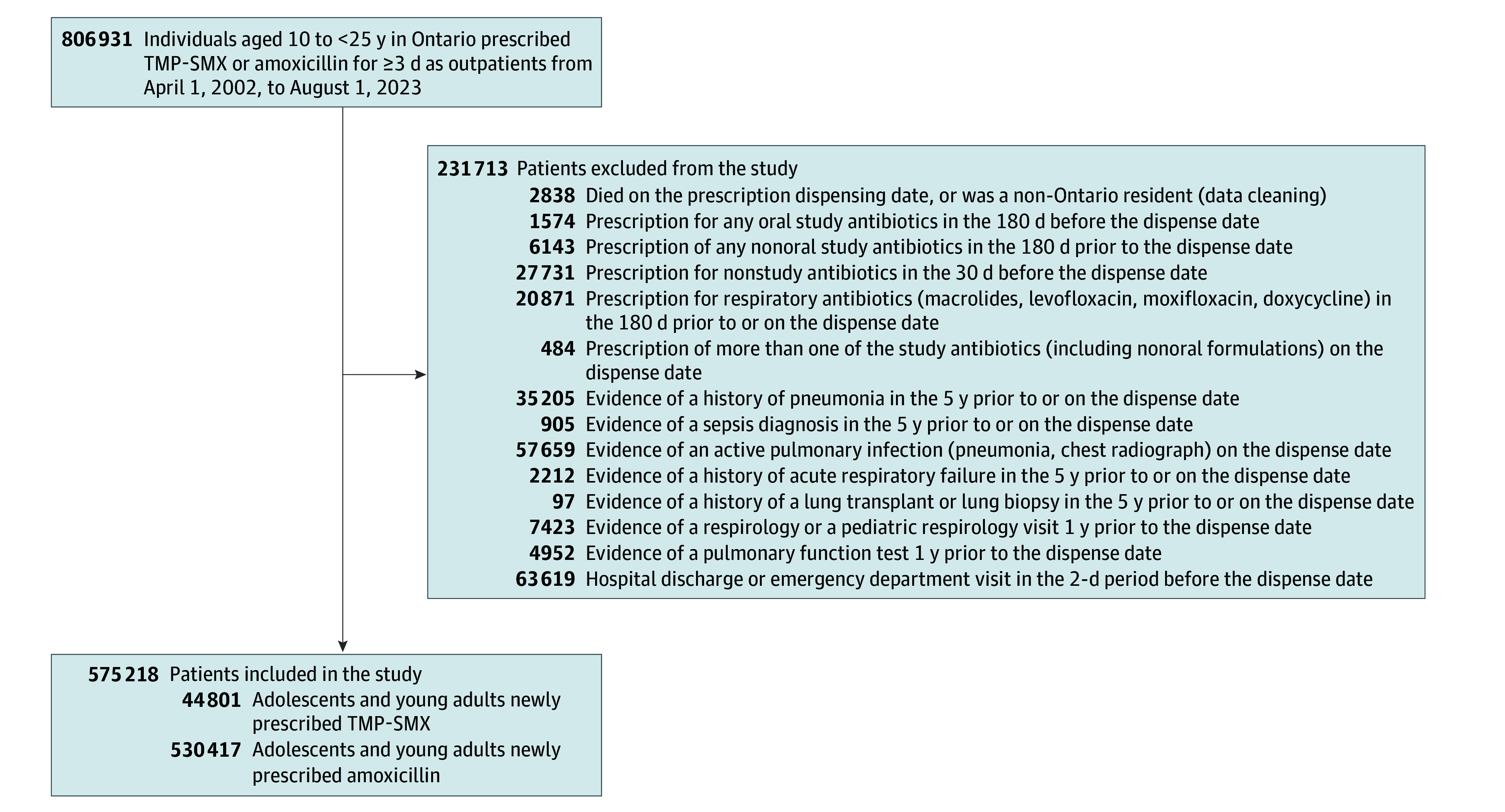
Flow Diagram of Cohort Build for TMP-SMX vs Amoxicillin TMP-SMX indicates trimethoprim-sulfamethoxazole.

The TMP-SMX vs amoxicillin cohort included 575 218 adolescents and young adults who were newly dispensed TMP-SMX (n = 44 801) or amoxicillin (n = 530 417) (median age after weighting, 19 years [IQR, 16-22 years]; 74.3% female) at an outpatient pharmacy (Table 1). In the amoxicillin group, 480 132 users (90.5%) were prescribed amoxicillin alone, whereas 50 285 (9.5%) were prescribed amoxicillin in combination with clavulanic acid. The flow diagram for the cohort build is shown in Figure 1.

**Table 1.** Baseline Characteristics of Adolescents and Young Adults Newly Prescribed TMP-SMX vs Those Newly Prescribed AMX in Ontario, Canada (2002-2023)^a^

Demographic characteristic	Unweighted data (n = 575 218)			Weighted data (n = 43 158)^b^		
	AMX (n = 530 417)	TMP-SMX (n = 44 801)	Standardized difference^c^	AMX (n = 21 579)	TMP-SMX (n = 21 579)	Standardized difference^c^
Sex, No. (%)						
Female	293 414 (55.3)	36 537 (81.6)	0.59	16 042 (74.3)	16 042 (74.3)	0
Male	237 003 (44.7)	8264 (18.4)	0.59	5536 (25.7)	5536 (25.7)	0
Age, mean (SD), y	18.2 (3.9)	18.8 (3.8)	0.16	18.6 (3.9)	18.6 (4.0)	0
Residence, No. (%)						
Urban	488 896 (92.2)	39 571 (88.3)	0.13	19 134 (88.7)	19 134 (88.7)	0
Rural	39 919 (7.5)	5078 (11.3)	0.13	2445 (11.3)	2445 (11.3)	0
Income quintile, No. (%)^d^						
1 (Lowest)	149 281 (28.1)	12 937 (28.9)	0.02	6732 (31.2)	6732 (31.2)	0
2	106 760 (20.1)	9238 (20.6)	0.01	4498 (20.8)	4498 (20.8)	0
3 (Middle)	97 372 (18.4)	8164 (18.2)	0.01	3886 (18.0)	3886 (18.0)	0
4	90 229 (17.0)	7358 (16.4)	0.02	3416 (15.8)	3416 (15.8)	0
5 (Highest)	84 785 (16.0)	6907 (15.4)	0.02	3047 (14.1)	3047 (14.1)	0
Prescriber, No. (%)						
Primary care physician	294 229 (55.5)	35 651 (79.6)	0.53	15 383 (71.3)	15 383 (71.3)	0
Dermatologist	103 (0.02)	142 (0.3)	0.08	32 (0.1)	32 (0.1)	0
Other	13 493 (2.5)	2138 (4.8)	0.12	1164 (5.4)	1164 (5.4)	0
Missing	222 592 (42.0)	6870 (15.3)	0.62	4999 (23.2)	4999 (23.2)	0
Comorbidities, No. (%)^e^						
Obesity	22 783 (4.3)	1857 (4.1)	0.01	982 (4.5)	982 (4.5)	0
Alcohol misuse	7448 (1.4)	947 (2.1)	0.05	474 (2.2)	474 (2.2)	0
Urinary tract infection	96 405 (18.2)	22 620 (50.5)	0.72	8473 (39.3)	8473 (39.3)	0
Skin or soft tissue infection	85 865 (16.2)	9385 (20.9)	0.12	4595 (21.3)	4595 (21.3)	0
Acne vulgaris	81 843 (15.4)	8085 (18.0)	0.07	3538 (16.4)	3538 (16.4)	0
Acne treatment	12 443 (2.3)	1172 (2.6)	0.02	533 (2.5)	533 (2.5)	0
Ear, nose, and throat infection	361 798 (68.2)	31 168 (69.6)	0.03	14 805 (68.6)	14 805 (68.6)	0
Charlson Comorbidity Index, mean (SD)^f^	0.01 (0.1)	0.04 (0.3)	0.12	0.02 (0.2)	0.02 (0.2)	0
Health care visits or tests^g^						
Primary care visits, mean (SD)	4.0 (5.5)	5.8 (7.3)	0.28	5.6 (7.2)	5.6 (7.7)	0
Emergency department visits, mean (SD)	0.5 (1.3)	0.9 (1.8)	0.23	0.9 (1.9)	0.9 (1.8)	0
Medication use, No. (%)^h^						
Benzodiazepines	7866 (1.5)	1096 (2.4)	0.07	503 (2.3)	503 (2.3)	0
Beta agonist and combinations	18 349 (3.5)	1695 (3.8)	0.02	877 (4.1)	877 (4.1)	0
Antifungals	2294 (0.4)	582 (1.3)	0.10)	212 (1.0)	212 (1.0)	0
Recent infections, No. (%)^i^						
Urinary tract infection	7448 (1.4)	22 664 (50.6)	1.35	4974 (23.1)	4974 (23.1)	0
Skin or soft tissue infection	7516 (1.4)	2035 (4.5)	0.18	1530 (7.1)	1530 (7.1)	0
Other infections	38 176 (7.2)	2288 (5.1)	0.09	1308 (6.1)	1308 (6.1)	0
Acne vulgaris	1822 (0.3)	778 (1.7)	0.14	286 (1.3)	286 (1.3)	0
Ear, nose, and throat infection	221 393 (41.7)	2401 (5.4)	0.95	2281 (10.6)	2281 (10.6)	0
Recent tests, No. (%)^j^						
Throat swab	7852 (1.5)	235 (0.5)	0.10	187 (0.9)	187 (0.9)	0
Wound swab	22 540 (4.2)	778 (1.7)	0.15	589 (2.7)	589 (2.7)	0
Complete blood count test	10 663 (2.0)	1691 (3.8)	0.11	988 (4.6)	988 (4.6)	0
Urine culture	18 152 (3.4)	24 857 (55.5)	1.39	6915 (32.0)	6915 (32.0)	0

Abbreviations: AMX, amoxicillin; TMP-SMX, trimethoprim-sulfamethoxazole.

^a^ Unless otherwise specified, baseline characteristics were assessed on the date the patient filled the study antibiotics prescription—the index date.

^b^ Weighted using overlap weighting based on propensity scores. The propensity score was estimated using multivariable logistic regression with 66 covariates chosen a priori (eTable 6 in Supplement 1).

^c^ Difference between groups divided by pooled SD; a value greater than 0.1 is interpreted as a meaningful difference.^16^

^d^ Income was categorized into fifths of average neighborhood income on the index date.

^e^ Baseline comorbidities were assessed in the 5-year period before the index date.

^f^ Calculated based on hospital admission data in the 3-year period before the index date; “no hospital admissions” received a score of 0.

^g^ Total number of health care visits or tests in the 12-month period before the index date.

^h^ Examined in the 120-day period before the index date (the Ontario Drug Benefit program dispenses a maximum 100-day supply).

^i^ Recent infections were assessed in the 7 days before the index date.

^j^ Recent tests were assessed in the 7 days before the index date.

After weighting, we had a pseudopopulation of 21 579 individuals in each group of TMP-SMX and amoxicillin users (Table 1). In the weighted cohort, standardized differences for all 89 variables were less than 10% (eTable 8 in Supplement 1). The median age in the weighted cohort was 19 years (IQR, 16-22 years), and 16 042 (74.3%) were female; 15 383 patients (71.3%) received prescriptions for TMP-SMX or amoxicillin primarily from primary care physicians. The median duration of continuous dispensing was 7 days (IQR, 3-7 days) for TMP-SMX and 7 days (IQR, 7-10 days) for amoxicillin.

#### TMP-SMX vs Cephalosporins Cohort

**Figure 2.**
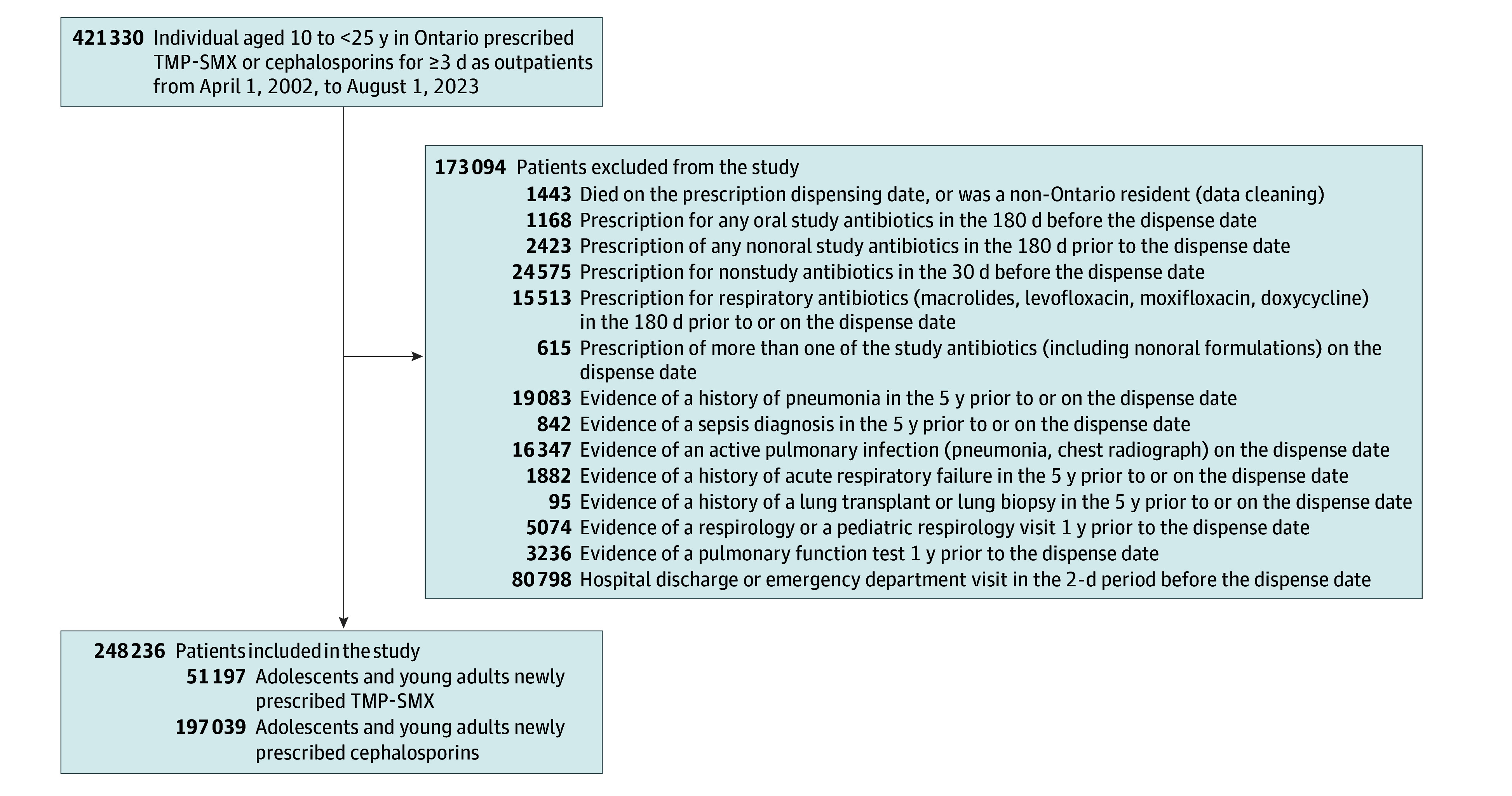
Flow Diagram of Cohort Build for TMP-SMX vs Cephalosporins Study TMP-SMX indicates trimethoprim-sulfamethoxazole.

The TMP-SMX vs cephalosporins cohort included 248 236 adolescents and young adults who were newly dispensed TMP-SMX (n = 51 197) or cephalosporins (n = 197 039) at an outpatient pharmacy (Table 2). In the cephalosporin group, 147 443 (74.8%) received cephalexin and 34 602 (17.6%) received cefprozil, the most prescribed medications. The flow diagram for the cohort build is shown in Figure 2.

**Table 2.** Baseline Characteristics of Adolescents and Young Adults Newly Prescribed TMP-SMX vs Those Newly Prescribed CEP in Ontario, Canada (2002-2023)^a^

Demographic Characteristic	Unweighted data (n = 248 236)			Weighted data (n = 41 076)^b^		
	CEP (n = 197 039)	TMP-SMX (n = 51 197)	Standardized difference^c^	CEP (n = 20 538)	TMP-SMX (n = 20 538)	Standardized difference^c^
Sex, No. (%)						
Female	106 156 (53.9)	42 267 (82.6)	0.65	14 847 (72.3)	14 847 (72.3)	0
Male	90 883 (46.1)	8930 (17.4)	0.65	5691 (27.7)	5691 (27.7)	0
Age, mean (SD), y	18.25 (4.0)	18.9 (3.8)	0.18	18.7 (3.9)	18.7 (4.0)	0
Residence, No. (%)						
Urban	182 187 (92.5)	45 404 (88.7)	0.13	18 336 (89.3)	18 336 (89.3)	0
Rural	14 270 (7.2)	5624 (11.0)	0.13	2202 (10.7)	2 202 (10.7)	0
Income quintile, No. (%)^d^						
1 (Lowest)	56 182 (28.5)	15 328 (29.9)	0.03	6396 (31.1)	6396 (31.1)	0
2	39 966 (20.3)	10 496 (20.5)	0.00	4252 (20.7)	4252 (20.7)	0
3 (Middle)	36 140 (18.3)	9232 (18.0)	0.01	3704 (18.0)	3704 (18.0)	0
4	33 019 (16.8)	8236 (16.1)	0.02	3268 (15.9)	3268 (15.9)	0
5 (Highest)	30 994 (15.7)	7679 (15.0)	0.02	2918 (14.2)	2918 (14.2)	0
Prescriber, No. (%)						
Primary care physician	164 506 (83.5)	40 779 (79.7)	0.10	15 797 (76.9)	15 797 (76.9)	0
Dermatologist	1136 (0.6)	152 (0.3)	0.04	74 (0.4)	74 (0.4)	0
Other	11 128 (5.6)	2432 (4.8)	0.04	1330 (6.5)	1330 (6.5)	0
Missing	20 269 (10.3)	7834 (15.3)	0.15	3338 (16.3)	3338 (16.3)	0
Comorbidities, No. (%)^e^						
Obesity	10 208 (5.2)	2195 (4.3)	0.04	989 (4.8)	989 (4.8)	0
Alcohol misuse	3267 (1.7)	1093 (2.1)	0.03	450 (2.2)	450 (2.2)	0
Urinary tract infection	40 258 (20.4)	26 291 (51.4)	0.68	7780 (37.9)	7780 (37.9)	0
Skin or soft tissue infection	47 738 (24.2)	9890 (19.3)	0.12	4433 (21.6)	4433 (21.6)	0
Acne vulgaris	34 463 (17.5)	9109 (17.8)	0.01	3487 (17.0)	3487 (17.0)	0
Acne treatment	5577 (2.8)	1389 (2.7)	0.01	552 (2.7)	552 (2.7)	0
Ear, nose, and throat infection	138 311 (70.2)	36 846 (72.0)	0.04	14 563 (70.9)	14 563 (70.9)	0
Charlson Comorbidity Index, mean (SD)^f^	0.01 (0.2)	0.04 (0.3)	0.11	0.03 (0.3)	0.03 (0.3)	0
Health care visits or tests^g^						
Primary care visits, mean (SD)	4.6 (6.0)	5.9 (7.4)	0.19	5.7 (7.5)	5.7 (7.7)	0
Emergency department visits, mean (SD)	0.6 (1.5)	0.9 (1.8)	0.16	0.9 (1.8)	0.9 (1.8)	0
Medication use, No. (%)^h^						
Benzodiazepines	3664 (1.9)	1300 (2.5)	0.04	508 (2.5)	508 (2.5)	0
Beta agonist and combinations	7599 (3.9)	2094 (4.1)	0.01	891 (4.3)	891 (4.3)	0
Antifungals	1133 (0.6)	727 (1.4)	0.08	217 (1.1)	217 (1.1)	0
Other antibiotics	4047 (2.1)	1836 (3.6)	0.09	638 (3.1)	638 (3.1)	0
Recent infections, No. (%)^i^						
Urinary tract infection	5317 (2.7)	26 393 (51.6)	1.32	4133 (10.1)	4133 (10.1)	0
Skin or soft tissue infection	56 351 (28.6)	2057 (4.0)	0.71	1956 (9.5)	1956 (9.5)	0
Other infections	5574 (2.8)	2606 (5.1)	0.12	1205 (5.9)	1205 (5.9)	0
Acne vulgaris	4170 (2.1)	856 (1.7)	0.03	430 (2.1)	430 (2.1)	0
Ear, nose, and throat infection	35 119 (17.8)	2737 (5.3)	0.40	2286 (11.1)	2286 (11.1)	0
Recent tests, No. (%)^j^						
Throat swab	1141 (0.6)	255 (0.5)	0.01	136 (0.7)	136 (0.7)	0
Wound swab	6210 (3.2)	813 (1.6)	0.10	595 (2.9)	595 (2.9)	0
Complete blood count test	5506 (2.8)	1935 (3.8)	0.06	877 (4.3)	877 (4.3)	0
Urine culture	9854 (5.0)	28 876 (56.4)	1.34	5543 (27.0)	5543 (27.0)	0

Abbreviations: CEP, cephalosporins; TMP-SMX, trimethoprim-sulfamethoxazole.

^a^ Unless otherwise specified, baseline characteristics were assessed on the date the patient filled the study antibiotics prescription—the index date.

^b^ Weighted using overlap weighting based on propensity scores. The propensity score was estimated using multivariable logistic regression with 66 covariates chosen a priori (eTable 6 in Supplement 1).

^c^ Difference between groups divided by pooled SD; a value greater than 0.1 is interpreted as a meaningful difference.^16^

^d^ Income was categorized into fifths of average neighborhood income on the index date.

^e^ Baseline comorbidities were assessed in the 5-year period before the index date.

^f^ Calculated based on hospital admission data in the 3-year period before the index date; “no hospital admissions” received a score of 0.

^g^ Total number of health care visits or tests in the 12-month period before the index date.

^h^ Examined in the 120-day period before the index date (the Ontario Drug Benefit program dispenses a maximum 100-day supply).

^i^ Recent infections were assessed in the 7 days before the index date.

^j^ Recent tests were assessed in the 7 days before the index date.

After weighting, we had a pseudopopulation 20 538 individuals in each group of TMP-SMX and cephalosporin users (Table 2). In the weighted cohort, the standardized differences for all 89 variables were less than 10% (eTable 9 in Supplement 1). The median age in the weighted cohort was 19 years (IQR, 16-22 years), and 14 847 (72.3%) were female; 15 797 (76.9%) received prescriptions for TMP-SMX or cephalosporins primarily from primary care physicians. The median duration of continuous dispensing was 7 days (IQR, 3-7 days) for TMP-SMX and 7 days (IQR, 7-10 days) for cephalosporins.

### Study Outcomes

#### TMP-SMX vs Amoxicillin Cohort

The primary outcome, a 30-day hospital visit with acute respiratory failure, occurred in 15 of 44 801 patients (0.03%) who started TMP-SMX and in 49 of 530 417 patients (0.01%) who started amoxicillin (number of weighted events, 7 of 21 579 [0.03%] for TMP-SMX and 2 of 21 579 [0.01%] for amoxicillin; weighted risk ratio, 2.79 [95% CI, 1.01-7.71]; weighted risk difference, 0.02% [95% CI, 0.001-0.04]) (Table 3). Among patients who experienced the outcome, the median time from starting the prescription to the outcome was 9 days (IQR, 3-19 days) in the TMP-SMX group and 20 days (IQR, 8-20 days) in the amoxicillin group. The number of events for the components of the composite outcome (ie, acute respiratory failure diagnosis and mechanical ventilation, ECMO, or tracheotomy) and the risk of all-cause hospitalization and all-cause mortality are shown in eTable 10 in Supplement 1. These results were confirmed using the negative control outcome (eTable 11 in Supplement 1).

**Table 3.** Risk of a Hospital Visit With Acute Respiratory Failure Diagnosis, Mechanical Ventilation, Tracheotomy, or ECMO in Adolescents and Young Adults Within 30 Days of Starting a New Prescription for TMP-SMX vs AMX and CEP

Primary outcome	Events, No./total No. (%)				RD, % (95% CI)	RR (95% CI)	Events, No./total No. (%)				RD, % (95% CI)	RR (95% CI)
	Unweighted		Weighted^a^				Unweighted		Weighted^a^			
	TMP-SMX	AMX	TMP-SMX	AMX			TMP-SMX	CEP	TMP-SMX	CEP		
Composite outcome^b^	15/44 801 (0.03)	49/530 417 (0.01)	7/21 579 (0.03)	2/21 579 (0.01)	0.02 (0.001-0.04)	2.79 (1.01-7.71)	17/51 197 (0.03)	21/197 039 (0.01)	8/20 538 (0.04)	3/20 538 (0.01)	0.02 (0.005-0.05)	2.85 (1.11-7.31)

Abbreviations: AMX, amoxicillin; CEP, cephalosporins; ECMO, extracorporeal membrane oxygenation; RD, risk difference; RR, risk ratio; TMP-SMX, trimethoprim-sulfamethoxazole.

^a^ Overlap weighting was used to balance comparison groups on indicators of baseline health. The propensity score (PS) was estimated using multivariable logistic regression with 84 covariates chosen a priori (eTable 6 in Supplement 1). Overlap weighting allocates weights to patients in proportion to the likelihood that they belong to the opposite treatment group. Treated patients are weighted by the probability of not being treated (1 − PS), while untreated patients are weighted by the probability of being treated (PS).^23^ Weighted RRs and 95% CIs were obtained through log-binomial regression, and weighted RDs and 95% CIs were obtained using binomial regression with an identity link function.

^b^ Hospital visit with acute respiratory failure diagnosis, mechanical ventilation, tracheotomy, or ECMO.

#### TMP-SMX vs Cephalosporins Cohort

The primary outcome, the 30-day hospital visit with acute respiratory failure, occurred in 17 of 51 197 patients (0.03%) who started TMP-SMX and in 21 of 197 039 patients (0.01%) who started cephalosporins (number of weighted events, 8 of 20 538 [0.04%] for TMP-SMX and 3 of 20 538 [0.01%] for cephalosporins; weighted risk ratio, 2.85 [95% CI, 1.11-7.31]; weighted risk difference, 0.02% [95% CI, 0.005%-0.05%]) (Table 3). Among patients who experienced the outcome, the median time from starting the prescription to the outcome was 9 days (IQR, 6-13 days) in the TMP-SMX group and 9 days (IQR, 2-17 days) in the cephalosporins group. The number of events for the components of the composite outcome (ie, acute respiratory failure diagnosis, mechanical ventilation, ECMO, or tracheotomy) and the risk of all-cause hospitalization and all-cause mortality are shown in eTable 10 in Supplement 1. These results were confirmed using the negative control outcome (eTable 11 in Supplement 1).

### Post Hoc Analyses

Results were consistent when the data were analyzed using a case-crossover analysis. A flow diagram of the cohort built for case-crossover analysis is provided in eFigure 2 in Supplement 1. Among 12 053 cases of acute respiratory failure included in the analysis, TMP-SMX use was associated with a higher risk of a hospital visit with acute respiratory failure (odds ratio, 1.45 [95% CI, 1.12-1.86]) (eTable 12 in Supplement 1).

## Discussion

In this cohort study of adolescents and young adults aged 10 and younger than 25 years, those who started a prescription for TMP-SMX had a significantly greater risk of hospitalization with acute respiratory failure compared with those who started amoxicillin or cephalosporins. Results were consistent in multiple sensitivity analyses and when using an alternative study design.

This population-based study of over 750 000 adolescents and young adults aged 10 to younger than 25 years in each cohort supported and reinforced the 2019 FDA safety warning and label change regarding a potential association between TMP-SMX and acute respiratory failure in healthy adolescents and young adults. Our findings confirmed and extended previous evidence, including over 19 case reports linking TMP-SMX to acute respiratory failure in adolescents and young adults. Notably, many of the cases in these reports required invasive ventilation or lung transplants, and respiratory issues often persisted after discontinuing the drug, sometimes leading to death^3,30,31^ (eTable 2 in Supplement 1).

Most patients prescribed TMP-SMX do not develop acute respiratory failure, but a small subset may be genetically predisposed. Variants in immune genes (eg, HLA-B07:02 and HLA-C07:02) and polymorphisms in drug-metabolizing enzymes could impair drug metabolism, leading to toxic intermediates and severe reactions. Alternatively, acute respiratory failure or acute respiratory distress syndrome may result from the underlying infection itself, rather than the drug. In this study, we tried to create comparison groups that were not significantly different in terms of infection status, tests for confirming infections, and anti-infective use by using overlap weighting (eTables 8 and 9 in Supplement 1).

This is the first population-based study, to our knowledge, to examine the association between TMP-SMX use and respiratory failure in adolescents and young adults aged 10 and younger than 25 years. The study was conducted in the usual clinical care setting, and overlap weighting was used to ensure that comparison groups were similar in all measured baseline characteristics. Several sensitivity analyses were conducted, and all supported our main findings. In particular, a case-crossover design was used to account for time-invariant confounders, including genetic factors.

### Limitations

This study has several limitations. First, the observational study design precludes reaching causal conclusions about the association between TMP-SMX use and acute respiratory failure, especially considering that infections themselves may lead to this complication. Therefore, the study requires replication before definitive conclusions about the association are reached. However, we excluded individuals with evidence of an active pulmonary infection on the index date, and after overlap weighting, baseline characteristics were similar between the groups of comparison for various types of infections, including urinary tract infections; skin or soft tissue infections; ear, nose, and throat infections; and other infections such as methicillin-resistant *Staphylococcus aureus* (MRSA). Nevertheless, we remain cognizant that residual confounding cannot be ruled out. Second, our study did not investigate longer durations of TMP-SMX use, such as those common in acne treatment (median duration of 21 days), which are typically off-label indications. In our region, the median duration of TMP-SMX use was 7 days, which aligns with typical short-course prescriptions for acute infections. Extending the study to include longer durations, similar to those described in case reports, may reveal a greater number of events and potentially uncover greater risk. Third, despite the use of highly accurate information on dispensing, it was not possible to know the proportion of patients who took their pills as prescribed. Fourth, the individuals studied were younger than 25 years, which is consistent with the age group cited in the case reports that prompted the FDA warning on TMP-SMX use. As a result, the generalizability of the study findings to other age groups, particularly those at higher risk of respiratory failure—such as older adults or individuals with prior lung conditions—remains unknown. Further studies are needed to replicate these findings in at-risk populations.

Fifth, this study could not assess the full benefit-risk ratio of TMP-SMX use. However, the number needed to harm for acute respiratory failure was 4976 for the TMP-SMX vs amoxicillin cohort and 4046 for the TMP-SMX vs cephalosporins cohort. In other words, for every 4000 to 5000 individuals aged 10 to younger than 25 years prescribed TMP-SMX vs amoxicillin or cephalosporins, 1 would be hospitalized with acute respiratory failure. Although TMP-SMX use was associated with an increased risk of acute respiratory failure compared with amoxicillin or cephalosporins, the absolute risk appears to be low. However, current evidence suggests that genetic factors, such as variations in HLA-B07:02 and HLA-C07:02, may elevate the risk of this adverse event for certain individuals. Further research is needed to identify genetic variants that predispose patients to TMP-SMX–related respiratory failure, enabling personalized treatment and a better risk-benefit assessment.

Sixth, most of the cases of acute respiratory failure identified in this study were related to mechanical ventilation, ECMO, or tracheotomy, which serve as proxies for acute respiratory failure. However, because these events were captured using coding systems linked to remuneration, we expect this proxy outcome to be recorded with high accuracy, as fee-for-service codes generally have high sensitivity and specificity.^32^ Also, these study results may not apply to individuals younger than 25 years who have private prescription drug insurance. Seventh, we lacked genotypic data to assess genetic links to TMP-SMX–related respiratory failure. However, a case-crossover analysis accounting for genetic factors showed consistent results, even though effect estimates were reduced (eTable 12 in Supplement 1). This suggests risk persists, may be higher for genetically susceptible individuals, and highlights the need for genotyping. This may suggest that the risk of acute respiratory failure persists even after accounting for genetic factors and that the risk could be higher for individuals with genetic variations. These results highlight the need for genotyping to identify individuals at higher risk of acute respiratory failure.

Eighth, although we focused on all oral cephalosporins to ensure an adequate sample size, the cephalosporin class is heterogeneous with different spectra of activity, making not all of them suitable comparators to TMP-SMX. However, because 93% of patients received either cephalexin or cefprozil, both of which are commonly used to treat urinary tract, respiratory tract, and skin infections, we can conclude that the majority of our cephalosporin cohort was using medications that are directly comparable to TMP-SMX.

Ninth, because individuals may have transitioned from private insurance to Ontario Health Insurance Plus, we could not ascertain their prescription medication use prior to Ontario Health Insurance Plus eligibility. As a result, some prior antibiotic exposure or medication history may have been missed. However, baseline comorbidities and outcomes were ascertained from population-based administrative databases that capture all Ontario residents and, therefore, were not affected by insurance status.

Tenth, whereas lower respiratory infections were excluded to reduce bias, upper respiratory infections were considered part of baseline patient characteristics. Although generally mild and self-limiting, upper respiratory infections could still act as potential confounders. To address this, proxy measures such as ear, nose, and throat infections and throat swabs were incorporated. These variables were well balanced between the exposed and comparator groups (eTables 8 and 9 in Supplement 1), suggesting that residual confounding from upper respiratory infections is unlikely to have materially influenced our findings

Also, in our study, we could not ascertain the clinical indications for antibiotic prescriptions from administrative data. To mitigate confounding by indication, we incorporated proxy measures for infection within 7 days before the prescription, including recent diagnoses of urinary tract, skin and soft tissue, and ear, nose, and throat infections, as well as related laboratory or diagnostic tests. These proxies reflect the most common reasons for prescribing these antibiotics and were well balanced between the exposed and comparator groups (eTables 8 and 9 in Supplement 1).

## Conclusions

In this cohort study of healthy adolescents and young adults starting TMP-SMX, the 30-day risk of a hospital visit with respiratory failure was higher than for those starting with amoxicillin or cephalosporins. These findings support the FDA warning, warranting replication, careful risk-benefit evaluation, and updates to product monographs and prescribing guidelines.

## Notes

Supplement 1. **eTable 1.** Literature search **eTable 2.** Case reports and case series reporting acute respiratory failure in otherwise healthy adolescents and young adults aged 10 to <25 years **eTable 3.** Coding definitions for demographic and comorbid conditions **eTable 4.** Exclusion criteria of the cohort build **eTable 5.** Operating characteristics of hospital diagnosis codes used to define the primary and secondary outcomes **eTable 6.** Variables included in the propensity score model **eTable 7.** The description of the overlap-weighting method and case-crossover analysis **eTable 8.** Baseline characteristics of adolescents and young adults newly prescribed TMP-SMX versus those newly prescribed amoxicilin in Ontario, Canada (2002–2023) **eTable 9.** Baseline characteristics of adolescents and young adults newly prescribed TMP-SMX versus those newly prescribed cephalosporins in Ontario, Canada (2002–2023) **eTable 10.** Risk of secondary outcomes (hospital visit with acute respiratory failure diagnosis, mechanical ventilation, tracheotomy, or ECMO) in adolescents and young adults within 30 days of starting a new prescription for TMP-SMX vs amoxicillin and cephalosporins **eTable 11.** Risk of a hospital visit with receipt of a nuclear medicine procedure (negative control) in adolescents and young adults within 30 days of starting a new prescription for TMP-SMX vs amoxicillin and cephalosporins **eTable 12.** Results from case-crossover analysis **eFigure 1.** Study design diagram comparing the use of TMP-SMX versus amoxicillin or cephalosporins and the risk of acute respiratory failure in individuals aged 10 to <25 **eFigure 2.** Cohort built for case-crossover analysis
eReferences.

Supplement 2.
Data Sharing Statement
