# Supplementary material for: Trimethoprim-Sulfamethoxazole and Acute Respiratory Failure in Adolescents and Young Adults
Source: JAMA Netw Open. 2025 Nov 24;8(11):e2545251. doi: 10.1001/jamanetworkopen.2025.45251 (PMC12645330; doi:10.1001/jamanetworkopen.2025.45251)
Supplement: Supplement 1. — eTable 1. Literature search eTable 2. Case reports and case series reporting acute respiratory failure in otherwise healthy adolescents and young adults aged 10 to <25 years eTable 3. Coding definitions for demographic and comorbid conditions eTable 4. Exclusion criteria of the cohort build eTable 5. Operating characteristics of hospital diagnosis codes used to define the primary and secondary outcomes eTable 6. Variables included in the propensity score model eTable 7. The description of the overlap-weighting method and case-crossover analysis eTable 8. Baseline characteristics of adolescents and young adults newly prescribed TMP-SMX versus those newly prescribed amoxicilin in Ontario, Canada (2002–2023) eTable 9. Baseline characteristics of adolescents and young adults newly prescribed TMP-SMX versus those newly prescribed cephalosporins in Ontario, Canada (2002–2023) eTable 10. Risk of secondary outcomes (hospital visit with acute respiratory failure diagnosis, mechanical ventilation, tracheotomy, or ECMO) in adolescents and young adults within 30 days of starting a new prescription for TMP-SMX vs amoxicillin and cephalosporins eTable 11. Risk of a hospital visit with receipt of a nuclear medicine procedure (negative control) in adolescents and young adults within 30 days of starting a new prescription for TMP-SMX vs amoxicillin and cephalosporins eTable 12. Results from case-crossover analysis eFigure 1. Study design diagram comparing the use of TMP-SMX versus amoxicillin or cephalosporins and the risk of acute respiratory failure in individuals aged 10 to <25 eFigure 2. Cohort built for case-crossover analysis eReferences. [file jamanetwopen-e2545251-s001.pdf]

Supplemental Online Content

Ahmadi F, McArthur E, Garcia-Bournissen F, Rieder MJ, Muanda FT. Trimethoprim-sulfamethoxazole and acute respiratory failure in adolescents and young adults. *JAMA Netw Open*. 2025;8(11):e2545251. doi:10.1001/jamanetworkopen.2025.45251

eTable 1. Literature search

eTable 2. Case reports and case series reporting acute respiratory failure in otherwise healthy adolescents and young adults aged 10 to <25 years

eTable 3. Coding definitions for demographic and comorbid conditions

eTable 4. Exclusion criteria of the cohort build

eTable 5. Operating characteristics of hospital diagnosis codes used to define the primary and secondary outcomes

eTable 6. Variables included in the propensity score model

eTable 7. The description of the overlap-weighting method and case-crossover analysis

eTable 8. Baseline characteristics of adolescents and young adults newly prescribed TMP-SMX versus those newly prescribed amoxicilin in Ontario, Canada (2002–2023)

eTable 9. Baseline characteristics of adolescents and young adults newly prescribed TMP-SMX versus those newly prescribed cephalosporins in Ontario, Canada (2002–2023)

eTable 10. Risk of secondary outcomes (hospital visit with acute respiratory failure diagnosis, mechanical ventilation, tracheotomy, or ECMO) in adolescents and young adults within 30 days of starting a new prescription for TMP-SMX vs amoxicillin and cephalosporins

eTable 11. Risk of a hospital visit with receipt of a nuclear medicine procedure (negative control) in adolescents and young adults within 30 days of starting a new prescription for TMP-SMX vs amoxicillin and cephalosporins

eTable 12. Results from case-crossover analysis

eFigure 1. Study design diagram comparing the use of TMP-SMX versus amoxicillin or cephalosporins and the risk of acute respiratory failure in individuals aged 10 to <25

eFigure 2. Cohort built for case-crossover analysis

eReferences.

This supplemental material has been provided by the authors to give readers additional information about their work.

eTable 1.

eTable 1a. Literature search in Medline (1946 to October 28, 2024)

#	Query	Number of studies
1	(acute respiration insufficiency or acute respiratory insufficiency or respiratory acute insufficiency or acute respiratory failure or acute respiratory distress syndrome or ards).tw.	39,409
2	exp Respiratory Insufficiency/co, dt, ep, et, mo, th [Complications, Drug Therapy, Epidemiology, Etiology, Mortality, Therapy]	46,609
3	1 or 2	81,315
4	(co?trimoxazol? or septra or bactrim or trimethoprim or sulfamethoxazol?).tw.	28,768
5	exp Trimethoprim, Sulfamethoxazole Drug Combination/ad, ae, po, tu, to [Administration & Dosage, Adverse Effects, Poisoning, Therapeutic Use, Toxicity]	5,293
6	4 or 5	30,875
7	3 and 6	114
8	limit 7 to english language	85

eTable 1b. Literature search in Embase (1947 to October 25, 2024)

#	Query	Results from 12 Mar 2024
1	(acute respiration insufficiency or acute respiratory insufficiency or respiratory acute insufficiency or acute respiratory failure or acute respiratory distress syndrome or ards).tw.	62,386
2	exp acute respiratory failure/co, dt, si, th [Complication, Drug Therapy, Side Effect, Therapy]	5,873
3	1 or 2	65,270
4	(co?trimoxazol? or septra or bactrim or trimethoprim or sulfamethoxazol?).tw.	41,922
5	exp cotrimoxazole/ae, ct, ad, dt, to, tm [Adverse Drug Reaction, Clinical Trial, Drug Administration, Drug Therapy, Drug Toxicity, Unexpected Outcome of Drug Treatment]	41,927
6	4 or 5	75,850
7	3 and 6	475
8	limit 7 to english language	439

eTable 2. Case reports and case series reporting acute respiratory failure in otherwise healthy adolescents and young adults aged 10 to < 25 years

Author/year	Age	Sex	Indication for TMP/SMX use	Oral Dose	Onset of respiratory symptoms after the first exposure (days)	Treatment	Outcome
**Natterer 2022**[1]	11	M	Chronic staphylococcus osteomyelitis	360mg BD (17mg/kg/d)	23	DC TMP/SMX, mechanical ventilation, HFPV, steroids	Slowly recovered
**Ocasio 2024**[2]	15	M	Impetigo	NA	8 (treatment was completed on day 7)	Respiratory support with NIPPV, steroids	Survived with Pulmonary fibrosis
**Donnan 2024**[3]	18	F	Extended spectrum beta-lactamase Klebsiella aerogenes pyelonephritis	NA	17	Steroids, ECMO	Survived with lung transplant
**Khanal 2024**[4]	17	F	Acne	NA	30	Mechanical ventilation, steroids	Slowly recovered
**Miller 2019**[5]	16	F	Acne	NA	24	DC TMP/SMX, ECMO, MMF	Survived
	17	F	Acne	NA	17	DC TMP/SMX, Mechanical ventilation, steroids	Survived
	13	F	Acne	NA	25	DC TMP/SMX, ECMO, steroids, lung and heart transplant	Died

	18	M	Acne	NA	23	DC TMP/SMX, ECMO, steroids	Survived
	15	F	Urinary tract infection	NA	10	DC TMP/SMX, ECMO, steroids, other immunosuppressive agents, plasma exchange	Died
**Persaud 2022**[6]	23	F	Acne	NA	18	ECMO, Steroids	Died
**Muradashvili 2023**[7]	23	F	Ocular toxoplasmosis	NA	24	DC TMP/SMX, ECMO, steroids	Died
**Rubin 2022**[8]	19	M	Acne	NA	14	ECMO	Still on mechanical ventilation after 99 days but improving

Abbreviations: TMP/SMX, trimethoprim/sulfamethoxazole; BD, twice daily; HFPV, high-frequency percussive ventilation; ECMO, extracorporeal membrane oxygenation; NIPPV, nasal intermittent positive pressure ventilation; MMF, mycophenolate mofetil

eTable 3. Coding definitions for demographic and comorbid conditions

Characteristic	Database	Codes
**Demographics**		
Age	RPDB	
Sex	RPDB	
Location of residence – Rural status	Statistics Canada	
Year of cohort entry	ODB	
Socioeconomic Status (Neighbourhood Income Quintile)	Statistics Canada	
LHIN[a]	RPDB	LHIN
Prescriber	ODB	
ODB Plancode	ODB	
**Comorbidities (5 years prior the cohort entry date)**		
Depression	CIHI-DAD	ICD-9: 2962, 2963, 3000, 3002, 3003, 3004, 3091, 311<br>ICD-10: F063, F064, F320, F321, F322, F323, F328, F329, F330, F331, F332, F333, F334, F338, F339, F341, F400, F401, F402, F408, F409, F410, F411, F412, F413, F418, F419, F420, F421, F422, F428, F429, F430, F431
	OHIP	OHIP DX: 311
	OMHRS (DSM-IV)	29189, 29284, 29289, 29383, 29384, 29620, 29621, 29622, 29623, 29624, 29625, 29626, 29630, 29631, 29632, 29633, 29634, 29635, 29636, 30000, 30001, 30002, 30021, 30022, 30023, 30029, 30030, 30040, 30113
Bipolar disorder	CIHI-DAD	ICD-9: 2960, 2961, 2964, 2965, 2966, 2967, 2968<br>ICD-10: F300, F301, F302, F308, F309, F310, F311, F312, F313, F314, F315, F316, F317, F318, F319
	OHIP	OHIP DX: 296<br>OHIP FEE: Q020
	OMHRS (DSM-IV)	29600, 29601, 29602, 29603, 29604, 29605, 29606, 29640, 29641, 29642, 29643, 29644, 29645, 29646, 29650, 29651, 29652, 29653, 29654, 29655, 29656, 29660, 29661, 29662, 29663, 29664, 29665, 29666, 29670, 29680, 29689
Cancer	CIHI-DAD	ICD-9 : 150, 154, 155, 157, 162, 174, 175, 185, 203, 204, 205, 206, 207, 208<br>ICD10: 971, 980, 982, 984, 985, 986, 987, 988, 989, 990, 991, 993, C15, C18, C19, C20, C22, C25, C34, C50, C56, C61, C82, C83, C85, C91, C92, C93, C94, C95, D00, D05

Characteristic	Database	Codes
	OHIP	OHIP DX :203, 204, 205, 206, 207, 208, 150, 154, 155, 157, 162, 174, 175, 183
Atopic dermatitis or eczema	CIHI-DAD	ICD-9: 6918, 540<br>ICD10: L208, L209, B000
	OHIP	OHIP DX: 691, 692, 690
Diabetes	ODB	Insulins, oral antihyperglycemic agents
Schizophrenia	CIHI-DAD	ICD-9:<br>2950, 2951, 2952, 2953, 2954, 2955, 2956, 2957, 2958, 2959, 2970, 2971, 2972, 2973, 2978, 2979, 2980, 2981, 2983, 2984, 2988, 2989<br>ICD-10: F060, F062, F105, F107, F115, F117, F125, F127, F135, F137, F145, F147, F155, F157, F165, F167, F175, F177, F185, F187, F195, F197, F200, F201, F202, F203, F204, "F205", "F206, F208, F209, F220, F228, F229, F230, F231, F232, F233, F238, F239, F24, F250, F251, F252, F258, F259, F28, F29
	OHIP	OHIP dx: 291, 292, 295, 297, 298<br>OHIP fee: Q021
	OMHRS (DSM-IV)	DSM-IV (OMHRS): 29130, 29150, 29211, 29212, 29381, 29382, 29510, 29520, 29530, 29540, 29560, 29570, 29590, 29710, 29730, 29880, 29890
Alcohol misuse	CIHI-DAD	ICD-9: 303, 3050, 2652, 2911, 2912, 2913, 2915, 2918, 2919, 3575, 4255, 5353, 5710, 5711, 5712, 5713, 980, V113<br>ICD10: E24, E512, F10, G312, G621, G721, I426, K292, K70, K860, T510, X45, X65, Y15, Y573, Z502, Z714, Z721
	OHIP	OHIP dx: 303

Characteristic	Database	Codes
Urinary tract infection	CIHI-DAD	59010, 59000, 59080, 5902, 5909, 5950, 59589, 5959, 5970, 5990, 6010, 6011, 6012, 6013, 6031, 6040, 60490, 99665<br>ICD10: N10, N11, N12, N136, N151, N159, N160, N300, N308, N309, N340, N390, N410, N411, N412, N413, N431, N45
	OHIP	OHIP DX: 590, 595, 597, 599, 601, 603, 604
Skin or soft tissue infection	OHIP	OHIP DX: 680, 682, 683, 684, 685, 686
	CIHI-DAD	ICD-9: 680, 681, 682, 683, 684, 685, 686, 035<br><br>ICD-10: L00, L01, L02, L03, L04, L05, L06, L07, L08, T814
Acne vulgaris	OHIP	OHIP DX: 706
	CIHI-DAD	ICD-9: 706<br>ICD-10: L70
Acne treatment	ODB	
Sexually transmitted infections	OHIP	OHIP DX: 042, 043, 044, 097, 622, 098, 099, 053, 054, 078, 131
	CIHI-DAD	ICD-9: 042, 0911, 092, 093, 094, 095, 096, 097, 054, 0781, 043, 044, 099, 0794<br>ICD-10: B20, B21, B22, B23, B24, A51, A53, A60, A630, K628, N870, N890, N900, N871, N891, N901, D013, D06, D071, D072, N872, N892, N902, C53, C21, C51, C52, A54, A55, A56, A57, A58, A59, A63, A64
Dental caries and procedures	OHIP	OHIP FEE: Q631, S900, T809, T810, A903, W903, C903<br><br>OHIP DX: 521
	CIHI-DAD	ICD-9: 5210<br>ICD-10: K020, K021, K022, K023, K024, K028, K029
Gingivitis or periodontitis	OHIP	OHIP DX: 523
	CIHI-DAD	ICD-9: 5230, 5231, 5233, 5234, 5235, 5238, 5239<br>ICD-10: K050, K051, K052, K053, K054, K055, K056
Eye infection	OHIP	OHIP DX: 372, 373, 370, 374, 375, 377, 363, 364

Characteristic	Database	Codes
	CIHI-DAD	ICD-9: 3720, 3721, 3722, 3723, 3729, 3700, 3702, 3704, 3705, 3706, 3708, 3709, 3640, 3641, 3642, 3643, 3600, 3730, 3733, 3750, 3751, 3752, 3753, 3754, 3756, 3758, 3759, 3731, 3732<br>ICD-10: H10, H119, H16, H20, H440, H01, H04, H00
Infective endocarditis		ICD9: 4210, 4211, 4219, 4249, 1128, 4010<br>ICD10: I38, I398, I330, B376, I339, I101<br>OHIP DX: 390,391
Bone infection	OHIP	OHIP DX: 739, 731, 730, 711, 727, 9966
	CIHI-DAD	ICD-9: 7300, 7301, 7302, 7110, 7114, 7113, 7114<br>ICD-10: M86, M00, M01, M02, M03, T845
ENT infection	OHIP	OHIP DX: 380, 381, 382, 383, 384, 386, 387, 388, 460, 461, 034
	CIHI-DAD	ICD-9: 3801, 3802, 3801, 3800, 3803, 3804, 3805, 3808, 3809, 3810, 3811, 3812, 3813, 3814, 3820, 3821, 3822, 3823, 3824, 3829, 3820, 3815, 3816, 3817, 3818, 3819, 3830, 3831, 3832, 3838, 3839, 3853, 3842, 3840, 3841, 3848, 3849, 3850, 3851, 3852, 3853, 3858, 3859, 3831, 3858, 3870, 3871, 3872, 3878, 3879, 3860, 3861, 3862, 3868, 3869<br>ICD-10: H60, H61, H62, H65, H66, H67, H68, H69, H70, H71, H72, H73, H74, H75, H80, H81, H83, J00, J01, J02, J04, J05, J06
Rosacea	OHIP	OHIP DX: 695
	CIHI-DAD	ICD-9: 6953, 7048, 7058, 7048, 7049<br>ICD-10: L71, L72, L73

Characteristic	Database	Codes
Major surgery	OHIP	OHIP FEE: S073, S074, S075, S080, S081, S082, S083, S084, S085, S086, S087, S088, S089, S090, S091, S092, S093, S095, S096, S097, S098, S099, S100, S102, S117, S120, S121, S122, S123, S124, S125, S128, S129, S131, S132, S133, S134, S135, S137, S138, S139, S140, S149, S150, S151, S154, S155, S156, S157, S158, S159, S160, S161, S162, S164, S165, S166, S167, S168, S169, S170, S171, S172, S173, S174, S175, S176, S177, S178, S179, S180, S181, S182, S183, S184, S185, S187, S188, S189, S191, S192, S193, S194, S195, S196, S197, S199, S201, S202, S203, S204, S205, S206, S213, S214, S215, S216, S217, S218, R700, R701, R702, R703, R704, R705, R708, R712, R713, R714, R715, R716, R717, R718, R720, R721, R722, R723, R724, R725, R726, R727, R728, R729, R730, R733, R734, R735, R736, R737, R738, R741, R742, R743, R746, R747, R748, R749, R750, R754, R755, R756, R757, R758, R759, R760, R761, R762, R763, R764, R765, R768, R769, R770, R771, R772, R773, R774, R780, R783, R784, R785, R786, R787, R788, R789, R790, R791, R792, R794, R795, R796, R797, R798, R799, R800, R801, R802, R803, R804, R805, R806, R807, R808, R809, R810, R811, R812, R813, R814, R815, R816, R817, R830, R831, R832, R833, R855, R856, R857, R858, R859, R860, R861, R862, R863, R864, R865, R875, R876, R877, R880, R881, R882, R883, R920, R921, R922, R923, R924, R925, R926, R927, R928, R929, R930, R932, R933, R934, R935, R936, R937
Fracture	OHIP	OHIP FEE: F095, F096, F097, Z211, F014, F022, F023, F025, F026, F028, F030, F032, F033, F046, F024, F027, F031, Z203
	CIHI-DAD	ICD-9: 8200, 8201, 8208, 8209, 8202, 8203, 8210, 8211, 813, 812, 8056, 8057, 808<br>ICD-10: S720, S721, S722, S723, S52, S422, S321, S322, S324, S323, S325, S327, S328<br>CCI: 1VA73, 1VC73, 1VA74, 1VA53, 1VC74, 1VA80, 1VC73, 1VC74, 1VC03, 1VC80, 1TV73, 1TV74, 1TV03<br>CCP: 9104, 9124, 9054, 9114, 9134, 935, 936, 9104, 9124, 9054, 9114, 9134, 9101, 9121, 9141, 9111, 9131, 9052
Other bacterial infections	CIHI-DAD	ICD-9: 040, 041<br>ICD-10: A49, A48
	OHIP	OHIP dx: 040, 023, 030, 032, 033, 034, 035, 036, 037, 038, 039, 009, 787
Obesity	CIHI-DAD	ICD-9: 2780<br>ICD10: E660, E661, E662, E668, E669
	OHIP	OHIP DX: 278
Hypothyroidism	CIHI-DAD	ICD-9: 243, 2440, 2441, 2442, 2443, 2448, 2449<br>ICD-10: E030, E031, E032, E033, E034, E035, E038, E039, E890, E00, E01, E02
	OHIP	OHIP DX: 243, 244
**Medication use (120 days before cohort entry)**		
All medications	ODB	

Characteristic	Database	Codes
**Healthcare Use (1 year prior to the cohort entry date)**		
GP/FP visits,<br>Dermatologist visits,<br>Internist visits	OHIP<br>IPDB	Mainspeciality = "GP/FP" or "F.P./EMERGENCY MEDICINE", "DERMATOLOGY", "INTERNAL MEDICINE"
Number of any hospitalizations	CIHI-DAD	"ddate"
Number of any ER visits	NACRS	"regdate"
TSH	OHIP	OHIP FEE: G016, L341
Calcium tests	OHIP	OHIP FEE: L045, L046
Cholesterol test	OHIP	OHIP FEE: L117, L055, L056, L156
CBC test	OHIP	OHIP FEE: L393
CT head	OHIP	OHIP FEE: X188, X400, X401, X402, X405, X408
CT abdomen	OHIP	OHIP FEE: X126, X409, X410
Sputum swabs	OHIP	OHIP FEE: L629
Throat swab	OHIP	OHIP FEE: L640, L636
Nasal swab	OHIP	OHIP FEE: L715
Vaginal smear	OHIP	OHIP FEE: L625
GC culture and smear	OHIP	OHIP FEE: L627
Wound swab	OHIP	OHIP FEE: L628
Blood smear	OHIP	OHIP FEE: L624
Urine culture	OHIP	OHIP FEE: L253, L254, L255, L633, L634, L641, G009, G010
CT pelvis	OHIP	OHIP FEE: X128, X415, X416
Echocardiography	CIHI-DAD	CCP: 0282<br>CCI: 3IP30
	OHIP	OHIP FEE: G560, G561, G562, G566, G567, G568, G570, G571, G572, G574, G575, G576, G577, G578, G581
Holter monitoring	CIHI-DAD	CCP: 0354<br>CCI: 2HZ24JAKH
	OHIP	OHIP FEE: G311, G320, G647, G648, G649, G650, G651, G652, G653, G654, G655, G656, G657, G658, G659, G660, G661, G682, G683, G684, G685, G686, G687, G688, G689, G690, G692, G693
Cervical cancer screening	OHIP	OHIP FEE: E430, G365, G394, L713, L812
Influenza vaccination	OHIP	OHIP FEE: G590, G591
Hearing test	OHIP	OHIP FEE: G153, G154, G440, G441, G442, G443, G448, G450, G451, G452, G525, G526, G529, G530, G533, G815, G816

eTable 4. Exclusion criteria of the cohort build

To ensure that individuals were new users of the study antibiotics (i.e., TMP-SMX, amoxicillin or cephalosporins), those with any evidence of study antibiotics use (including combination drug prescriptions) in the 180 days before the index date were excluded. For each analysis, a distinct TMP-SMX cohort was constructed by excluding patients with prior use of either the study drug or the specific comparator in the 180 days before cohort entry. These analysis-specific exclusions result in different cohort sizes for TMP-SMX across comparisons. Those who were discharged from the hospital or emergency department within two days before the index date were also excluded (in Ontario, an individual who starts an antibiotic prescription during a hospital admission would have their outpatient prescription dispensed on the same day or the day after hospital discharge). To ensure that study antibiotics were the primary antibiotics started for the individual's current infection, those with any evidence of a non-study antibiotic prescription within 30 days prior to the index date were excluded. To ensure that individuals were as healthy as those described in prior case reports and had no prior lung conditions that could affect study outcomes, we excluded those with any pulmonary conditions, such as prior acute respiratory failure, acute pulmonary infection on the index date, prescriptions for respiratory antibiotics (macrolides, levofloxacin, moxifloxacin, doxycycline) within the previous 180 days, a history of pneumonia, sepsis, lung transplant, or lung biopsy within the past five years, or a respirology visit or pulmonary function test within the past year. All the exclusion criteria described above applied separately to the two cohorts: the TMP-SMX vs amoxicillin cohort and the TMP-SMX vs cephalosporins cohort

eTable 5. Operating characteristics of hospital diagnosis codes used to define the primary and secondary outcomes

Outcome	Outcome component	ICD-10/CCI/OHIP/ORGD codes in this study	ICD-10 codes used in the validation study	Reference Standard	Operating Characteristics, % (95% CI)			Study
					Sensitivity	Specificity	Positive predictive value	
Hospital visit with acute respiratory failure diagnosis, mechanical ventilation, tracheotomy, or ECMO	Acute respiratory failure diagnosis, Mechanical ventilation, ECMO, Tracheotomy	OHIP FEE: G558, G557, G405, G406, G407, Z741, Z325, Z788<br>ICD-10: J80,J96 Z930<br>CCI: 1LZ38,1LZ37, 1GZ31, 1GJ77, 1GJ55						
Hospital visit with receipt of a nuclear medicine procedure (negative control)		OHIP FEE : J850, J650, J852, J862						
**Death**					98	100		Jha P 1996[9]

Abbreviations: ICD-10, International Classification of Diseases, Tenth Revision; ECMO, extracorporeal membrane oxygenation; NA, not available

eTable 6. Variables included in the propensity score model

Category	Variables
**Demographics (on the index date)**	Age, sex, year of cohort entry, neighborhood income quintile, rural residence, Local Health Integration Network, prescriber type , ODB plan code
**Comorbidities (within five years prior to the index date)**	Obesity, alcohol misuse, bipolar disorder, diabetes, hypothyroidism, schizophrenia, depression, cancer, fracture, major surgery, eczema, urinary tract infection, skin or soft tissue infection, other infection, acne vulgaris, acne treatment, sexually transmitted infection, infective endocarditis, dental caries and procedures, gingivitis or periodontitis, eye infection, bone infection, rosacea, eczema, Charlson comorbidity index
**Recent infections (within seven days prior or on the index date)**	Urinary tract infection, skin infection, other infection, sexually transmitted infection, acne vulgaris, acne treatment, dental caries and procedures, gingivitis or periodontitis, eye infection, bone infection, rosacea, eczema, ear infection
**Medications (within 120 days prior to the index date)**	antifungals, H2 blockers, benzodiazepine, beta-agonists, glucocorticoid, opioids, antipsychotics, proton pump inhibitors, selective serotonin reuptake inhibitors, prescription NSAIDs, Estrogen and combinations, other antibiotics, number of unique drug names, number of days supplied
**Health Care Use and investigations (within one year prior to the index date)**	Emergency department visits, family physician visits, hospitalizations, dermatologist visits, internist visits, Throat swab, vaginal swab, GC smear, wound swab, urine culture, TSH test, cervical cancer screening, cholesterol test (total cholesterol, HDL), CT abdomen, CT head, CT pelvis, echocardiography, flu shot, hearing test, Holter monitoring, calcium test, CBC test
**Health Care Use and investigations (within seven days prior or on the index date)**	Throat swab, vaginal swab, GC smear, wound swab, urine culture, CBC test, OHIP Billing

Abbreviations: ENT infection, ear nose throat infection; CT, computed tomography; H2 blockers, Histamine H2-receptor antagonists; TSH test, thyroid-stimulating hormone test; CBC, complete blood count; NSAIDs, Non-steroidal anti-inflammatory drugs.

eTable 7. The description of the overlap-weighting method and case-crossover analysis.

Propensity score overlap-weighting

Overlap weighting was used to balance comparison groups on indicators of baseline health. The propensity score was estimated using multivariable logistic regression with 84 covariates chosen *a priori* (defined in eTable 6 in Supplement 1). These covariates were assessed within prespecified windows: comorbidities during the five years prior to the index date, medication use during the 120 days prior, health care use and investigations during the one year prior, and recent infections, health care use, and investigations within seven days prior to or on the index date. Covariates were selected based on their potential to act as confounders, known risk factors for the outcomes, or proxies for such risk factors, guided by prior literature and clinical relevance.[10] Overlap weighting assigns weights to patients based on their predicted probability of receiving the opposite treatment. Treated patients were weighted by the probability of not being treated (1 − PS), while untreated patients were weighted by the probability of being treated (PS). This approach minimizes the influence of extreme propensity score (PS) values, preventing outliers—those who are almost always treated (PS near 1) or never treated (PS near 0)—from skewing the results and reducing precision, as can occur with IPTW. Overlap weighting also has the advantage of achieving an exact covariate balance between the treated and reference groups by construction.[11] This method produces a weighted population with reasonable clinical equipoise for treatment decisions.[11,12] Between-group differences in baseline characteristics were compared using standardized differences (SD) in both the unweighted and weighted samples[13] (differences >10% were considered meaningful). Weighted risk ratios with 95% confidence intervals (CIs) were obtained using log-binomial regression [14], and weighted risk differences with 95% CIs were obtained using binomial regression with an identity link function.

Case-crossover analysis:

Case-crossover analysis is a design that studies the association of transient drug exposures with acute outcomes. Each participant in this design serves as their own control; therefore, characteristics that do not vary over time, such as sex, ethnicity, and genetic factors, cannot confound the analysis.[15] Briefly, we built a cohort of individuals who experienced the primary outcome within the study's accrual period from April 1, 2002, to August 1, 2023. The index date was defined as the date of occurrence of the primary outcome. The case period (i.e., the period at risk) was the 30-day period preceding the index date, during which we assessed the proportion of prescriptions for TMP-SMX. This was compared to the control period, which was defined as the 30 days from 61 to 90 days prior to the index date, where we also assessed the proportion of TMP-SMX prescriptions. To reduce potential confounding from prior medication use, we implemented a washout period from 31 to 60 days prior to the index date. This washout period ensured that any medication exposure during the case period (30 days before the index date) was not influenced by prescriptions or treatment during the preceding 31 to 60 days. The control period was presumed to be unrelated to the primary outcome. To estimate the association, we calculated the odds ratio (OR) by comparing TMP-SMX exposure between the case and control periods, using discordant pairs—instances where individuals were exposed in the case period but not in the control period or vice versa. The OR was calculated as the ratio of these discordant pairs.

eTable 8. Baseline characteristics of adolescents and young adults newly prescribed TMP-SMX versus those newly prescribed amoxicillin in Ontario, Canada (2002–2023)

Variable	Value	Pre-weighting					Post-weighting				
		amoxicillin		TMP-SMX		Stan. Diff.	amoxicillin		TMP-SMX		Stan. Diff.
		N=530,417		N=44,801			N=21,579		N=21,579		
		n	%	n	%		n	%	n	%	
**Demographics**											
Age	Mean ± SD	18.22	3.93	18.84	3.85	0.16	18.64	3.95	18.64	3.98	0.00
	Median (IQR)	18	(15-22)	19	(16-22)		19	(16-22)	19	(16-22)	
	10-14	107634	20.3%	6793	15.2%	0.13	3817	17.7%	3817	17.7%	0.00
	15-19	199715	37.7%	15925	35.5%	0.05	7438	34.5%	7438	34.5%	0.00
	20+	223068	42.1%	22083	49.3%	0.14	10323	47.8%	10323	47.8%	0.00
Sex	F	293414	55.3%	36537	81.6%	0.59	16042	74.3%	16042	74.3%	0.00
	M	237003	44.7%	8264	18.4%	0.59	5536	25.7%	5536	25.7%	0.00
Income quintile	Missing	1990	0.4%	197	0.4%	0.00	* imputed as '1'		* imputed as '1'		
	1	149281	28.1%	12937	28.9%	0.02	6732	31.2%	6732	31.2%	0.00
	2	106760	20.1%	9238	20.6%	0.01	4498	20.8%	4498	20.8%	0.00
	3	97372	18.4%	8164	18.2%	0.01	3886	18.0%	3886	18.0%	0.00
	4	90229	17.0%	7358	16.4%	0.02	3416	15.8%	3416	15.8%	0.00
	5	84785	16.0%	6907	15.4%	0.02	3047	14.1%	3047	14.1%	0.00
Rural	Missing	1602	0.3%	152	0.3%	0.00	* imputed as 'N'		* imputed as 'N'		
	N	488896	92.2%	39571	88.3%	0.13	19134	88.7%	19134	88.7%	0.00
	Y	39919	7.5%	5078	11.3%	0.13	2445	11.3%	2445	11.3%	0.00

Variable	Value	Pre-weighting					Post-weighting				
		amoxicillin		TMP-SMX		Stan. Diff.	amoxicillin		TMP-SMX		Stan. Diff.
		N=530,417		N=44,801			N=21,579		N=21,579		
		n	%	n	%		n	%	n	%	
ODB plancode	OTHER	33979	6.4%	3137	7.0%	0.02	1502	7.0%	1502	7.0%	0.00
	ODSP	56860	10.7%	6221	13.9%	0.10	3103	14.4%	3103	14.4%	0.00
	OW	80701	15.2%	9519	21.2%	0.16	4696	21.8%	4696	21.8%	0.00
	OHIP+	358877	67.7%	25924	57.9%	0.20	12278	56.9%	12278	56.9%	0.00
LHIN	1	31775	6.0%	3193	7.1%	0.04	1601	7.4%	1601	7.4%	0.00
	2	35748	6.7%	3690	8.2%	0.06	1710	7.9%	1710	7.9%	0.00
	3	30483	5.7%	2866	6.4%	0.03	1285	6.0%	1285	6.0%	0.00
	4	63223	11.9%	5694	12.7%	0.02	2773	12.9%	2773	12.9%	0.00
	5	44584	8.4%	2715	6.1%	0.09	1427	6.6%	1427	6.6%	0.00
	6	47654	9.0%	2582	5.8%	0.12	1329	6.2%	1329	6.2%	0.00
	7	39772	7.5%	2699	6.0%	0.06	1410	6.5%	1410	6.5%	0.00
	8	70702	13.3%	3998	8.9%	0.14	2003	9.3%	2003	9.3%	0.00
	9	67468	12.7%	4184	9.3%	0.11	2210	10.2%	2210	10.2%	0.00
	10	18153	3.4%	2332	5.2%	0.09	1090	5.1%	1090	5.1%	0.00
	11	41449	7.8%	4881	10.9%	0.11	2069	9.6%	2069	9.6%	0.00
	12	16523	3.1%	1882	4.2%	0.06	843	3.9%	843	3.9%	0.00
	13	17431	3.3%	2983	6.7%	0.16	1293	6.0%	1293	6.0%	0.00
	14	5452	1.0%	1102	2.5%	0.11	535	2.5%	535	2.5%	0.00
Year of entry	2002-2007	51853	9.8%	7540	16.8%	0.21	3602	16.7%	3602	16.7%	0.00
	2008-2013	63277	11.9%	6768	15.1%	0.09	3306	15.3%	3306	15.3%	0.00
	2014-2019	280465	52.9%	21091	47.1%	0.12	9466	43.9%	9466	43.9%	0.00
	2020+	134822	25.4%	9402	21.0%	0.10	5205	24.1%	5205	24.1%	0.00
Prescriber specialty	MISSING	222592	42.0%	6870	15.3%	0.62	4999	23.2%	4999	23.2%	0.00
	OTHER	13493	2.5%	2138	4.8%	0.12	1164	5.4%	1164	5.4%	0.00

Variable	Value	Pre-weighting					Post-weighting				
		amoxicillin		TMP-SMX		Stan. Diff.	amoxicillin		TMP-SMX		Stan. Diff.
		N=530,417		N=44,801			N=21,579		N=21,579		
		n	%	n	%		n	%	n	%	
	DERMATOLOGY	103	0.0%	142	0.3%	0.08	32	0.1%	32	0.1%	0.00
	PCP	294229	55.5%	35651	79.6%	0.53	15383	71.3%	15383	71.3%	0.00
**Medication use (120 days prior to the index date)**											
Number of days supplied	3-7	324250	61.1%	34932	78.0%	0.37	15632	72.4%	15632	72.4%	0.00
	8-14	203788	38.4%	7395	16.5%	0.51	5159	23.9%	5159	23.9%	0.00
	15+	2379	0.4%	2474	5.5%	0.30	788	3.7%	788	3.7%	0.00
Benzodiazepines		7866	1.5%	1096	2.4%	0.07	503	2.3%	503	2.3%	0.00
Antipsychotics		14078	2.7%	1734	3.9%	0.07	892	4.1%	892	4.1%	0.00
Opioids		10812	2.0%	1488	3.3%	0.08	716	3.3%	716	3.3%	0.00
Selective serotonin reuptake inhibitor		32250	6.1%	4284	9.6%	0.13	2002	9.3%	2002	9.3%	0.00
Corticoid and combinations		38529	7.3%	3613	8.1%	0.03	1813	8.4%	1813	8.4%	0.00
Estrogen and combinations		34957	6.6%	7061	15.8%	0.29	2483	11.5%	2483	11.5%	0.00
Proton pump inhibitor		8258	1.6%	1067	2.4%	0.06	524	2.4%	524	2.4%	0.00
Histamine H2 receptor antagonist		3004	0.6%	471	1.1%	0.05	234	1.1%	234	1.1%	0.00
Beta agonist and combinations		18349	3.5%	1695	3.8%	0.02	877	4.1%	877	4.1%	0.00
Immunosuppressive agents		16979	3.2%	1794	4.0%	0.04	878	4.1%	878	4.1%	0.00

Variable	Value	Pre-weighting					Post-weighting				
		amoxicillin		TMP-SMX		Stan. Diff.	amoxicillin		TMP-SMX		Stan. Diff.
		N=530,417		N=44,801			N=21,579		N=21,579		
		n	%	n	%		n	%	n	%	
Antifungal agent		2294	0.4%	582	1.3%	0.10	212	1.0%	212	1.0%	0.00
Other antibiotics		16536	3.1%	2725	6.1%	0.14	1245	5.8%	1245	5.8%	0.00
Number of unique drug names	Mean ± SD	0.76	1.32	1.31	1.71	0.36	1.21	1.7	1.21	1.69	0.00
	Median (IQR)	0	(0-1)	1	(0-2)		1	(0-2)	1	(0-2)	
	0	327203	61.7%	18788	41.9%	0.40	10033	46.5%	10033	46.5%	0.00
	1-2	155061	29.2%	18075	40.3%	0.23	8007	37.1%	8007	37.1%	0.00
	3-4	35591	6.7%	5560	12.4%	0.19	2457	11.4%	2457	11.4%	0.00
	5+	12562	2.4%	2378	5.3%	0.15	1082	5.0%	1082	5.0%	0.00
**Comorbidities (5 years prior to the index date)**											
Obesity		22783	4.3%	1857	4.1%	0.01	982	4.5%	982	4.5%	0.00
Diabetes mellitus		3815	0.7%	391	0.9%	0.02	201	0.9%	201	0.9%	0.00
Depression		52653	9.9%	6705	15.0%	0.15	3084	14.3%	3084	14.3%	0.00
Hypothyroidism		8857	1.7%	849	1.9%	0.02	423	2.0%	423	2.0%	0.00
Alcohol misuse		7448	1.4%	947	2.1%	0.05	474	2.2%	474	2.2%	0.00
Cancer		3343	0.6%	663	1.5%	0.09	244	1.1%	244	1.1%	0.00
Schizophrenia		10313	1.9%	1192	2.7%	0.05	621	2.9%	621	2.9%	0.00
Bipolar disorder		16819	3.2%	2176	4.9%	0.09	1031	4.8%	1031	4.8%	0.00
Urinary tract infection		96405	18.2%	22620	50.5%	0.72	8473	39.3%	8473	39.3%	0.00
Skin or soft tissue infection		85865	16.2%	9385	20.9%	0.12	4595	21.3%	4595	21.3%	0.00
Other infection		247749	46.7%	25257	56.4%	0.20	11869	55.0%	11869	55.0%	0.00
Acne vulgaris		81843	15.4%	8085	18.0%	0.07	3538	16.4%	3538	16.4%	0.00

Variable	Value	Pre-weighting					Post-weighting				
		amoxicillin		TMP-SMX		Stan. Diff.	amoxicillin		TMP-SMX		Stan. Diff.
		N=530,417		N=44,801			N=21,579		N=21,579		
		n	%	n	%		n	%	n	%	
Acne treatment		12443	2.3%	1172	2.6%	0.02	533	2.5%	533	2.5%	0.00
Sexually transmitted infection		92816	17.5%	11299	25.2%	0.19	4931	22.9%	4931	22.9%	0.00
Infective endocarditis		290	0.1%	27	0.1%	0.00	15	0.1%	15	0.1%	0.00
Dental caries and procedures		28369	5.3%	3069	6.9%	0.07	1483	6.9%	1483	6.9%	0.00
Gingivitis or periodontitis		3376	0.6%	294	0.7%	0.01	149	0.7%	149	0.7%	0.00
Eye infection		69110	13.0%	6307	14.1%	0.03	2967	13.7%	2967	13.7%	0.00
Bone infection		55509	10.5%	5272	11.8%	0.04	2513	11.6%	2513	11.6%	0.00
ENT infection		361798	68.2%	31168	69.6%	0.03	14805	68.6%	14805	68.6%	0.00
Rosacea		7361	1.4%	934	2.1%	0.05	431	2.0%	431	2.0%	0.00
Eczema		145022	27.3%	13877	31.0%	0.08	6516	30.2%	6516	30.2%	0.00
Major surgery		5168	1.0%	587	1.3%	0.03	273	1.3%	273	1.3%	0.00
Fracture		22461	4.2%	1530	3.4%	0.04	803	3.7%	803	3.7%	0.00
Charlson Comorbidity Index	Mean ± SD	0.01	0.12	0.04	0.34	0.12	0.02	0.25	0.02	0.25	0.00
	Median (IQR)	0	(0-0)	0	(0-0)		0	(0-0)	0	(0-0)	
	0	527721	99.5%	43919	98.0%	0.14	21288	98.7%	21288	98.7%	0.00
	1	1988	0.4%	293	0.7%	0.04	138	0.6%	138	0.6%	0.00
	2	548	0.1%	438	1.0%	0.12	115	0.5%	115	0.5%	0.00

Variable	Value	Pre-weighting					Post-weighting				
		amoxicillin		TMP-SMX		Stan. Diff.	amoxicillin		TMP-SMX		Stan. Diff.
		N=530,417		N=44,801			N=21,579		N=21,579		
		n	%	n	%		n	%	n	%	
	3+	160	0.0%	151	0.3%	0.08	38	0.2%	38	0.2%	0.00
**Healthcare visits/tests (365 days prior to the index date)**											
Primary care provider visits	Mean ± SD	3.97	5.54	5.77	7.35	0.28	5.56	7.24	5.56	7.68	0.00
	Median (IQR)	3	(1-5)	4	(2-7)		4	(2-7)	4	(2-7)	
	0	94266	17.8%	4200	9.4%	0.25	2501	11.6%	2501	11.6%	0.00
	1	87474	16.5%	5201	11.6%	0.14	2699	12.5%	2699	12.5%	0.00
	2	76610	14.4%	5431	12.1%	0.07	2658	12.3%	2658	12.3%	0.00
	3+	272067	51.3%	29969	66.9%	0.32	13721	63.6%	13721	63.6%	0.00
Hospitalizations	Mean ± SD	0.05	0.28	0.12	0.55	0.16	0.10	0.45	0.10	0.45	0.00
	Median (IQR)	0	(0-0)	0	(0-0)		0	(0-0)	0	(0-0)	
	0	509413	96.0%	41208	92.0%	0.17	19887	92.2%	19887	92.2%	0.00
	1	17887	3.4%	2770	6.2%	0.13	1358	6.3%	1358	6.3%	0.00
	2	2294	0.4%	494	1.1%	0.08	217	1.0%	217	1.0%	0.00
	3+	823	0.2%	329	0.7%	0.07	116	0.5%	116	0.5%	0.00
ED visits	Mean ± SD	0.53	1.29	0.9	1.82	0.23	0.90	1.89	0.90	1.81	0.00
	Median (IQR)	0	(0-1)	0	(0-1)		0	(0-1)	0	(0-1)	
	0	380408	71.7%	27021	60.3%	0.24	13079	60.6%	13079	60.6%	0.00
	1	89447	16.9%	9050	20.2%	0.08	4257	19.7%	4257	19.7%	0.00
	2	31856	6.0%	3941	8.8%	0.11	1890	8.8%	1890	8.8%	0.00
	3+	28706	5.4%	4789	10.7%	0.20	2353	10.9%	2353	10.9%	0.00
Dermatologist visits	Mean ± SD	0.08	0.69	0.08	0.59	0.00	0.08	0.6	0.08	0.6	0.00
	Median (IQR)	0	(0-0)	0	(0-0)		0	(0-0)	0	(0-0)	
	0	509347	96.0%	42896	95.7%	0.02	20691	95.9%	20691	95.9%	0.00

Variable	Value	Pre-weighting					Post-weighting				
		amoxicillin		TMP-SMX		Stan. Diff.	amoxicillin		TMP-SMX		Stan. Diff.
		N=530,417		N=44,801			N=21,579		N=21,579		
		n	%	n	%		n	%	n	%	
	1	13354	2.5%	1168	2.6%	0.01	552	2.6%	552	2.6%	0.00
	2	3675	0.7%	366	0.8%	0.01	162	0.8%	162	0.8%	0.00
	3+	4041	0.8%	371	0.8%	0.00	174	0.8%	174	0.8%	0.00
Internist visits	Mean ± SD	0.09	0.56	0.16	1.15	0.08	0.14	0.88	0.14	0.92	0.00
	Median (IQR)	0	(0-0)	0	(0-0)		0	(0-0)	0	(0-0)	
	0	502450	94.7%	41474	92.6%	0.09	20002	92.7%	20002	92.7%	0.00
	1	19470	3.7%	2113	4.7%	0.05	1029	4.8%	1029	4.8%	0.00
	2	4638	0.9%	609	1.4%	0.05	281	1.3%	281	1.3%	0.00
	3+	3859	0.7%	605	1.4%	0.07	266	1.2%	266	1.2%	0.00
CT abdomen		3718	0.7%	799	1.8%	0.10	347	1.6%	347	1.6%	0.00
CT head		8217	1.5%	1088	2.4%	0.07	495	2.3%	495	2.3%	0.00
CT pelvis		3717	0.7%	794	1.8%	0.10	349	1.6%	349	1.6%	0.00
Echocardiography		8553	1.6%	1018	2.3%	0.05	456	2.1%	456	2.1%	0.00
Holter monitoring		5043	1.0%	566	1.3%	0.03	269	1.2%	269	1.2%	0.00
Cervical cancer screening		30958	5.8%	6322	14.1%	0.28	2756	12.8%	2756	12.8%	0.00
Influenza vaccination		31411	5.9%	2789	6.2%	0.01	1380	6.4%	1380	6.4%	0.00
Hearing test		5364	1.0%	496	1.1%	0.01	258	1.2%	258	1.2%	0.00
TSH test		106008	20.0%	10707	23.9%	0.09	5316	24.6%	5316	24.6%	0.00
Calcium test		11570	2.2%	1444	3.2%	0.06	683	3.2%	683	3.2%	0.00
Cholesterol test		49724	9.4%	4263	9.5%	0.00	2174	10.1%	2174	10.1%	0.00
CBC test		139433	26.3%	14676	32.8%	0.14	7305	33.9%	7305	33.9%	0.00

Variable	Value	Pre-weighting					Post-weighting				
		amoxicillin		TMP-SMX		Stan. Diff.	amoxicillin		TMP-SMX		Stan. Diff.
		N=530,417		N=44,801			N=21,579		N=21,579		
		n	%	n	%		n	%	n	%	
**Recent tests**											
Throat swab, within 7 days		7852	1.5%	235	0.5%	0.10	187	0.9%	187	0.9%	0.00
Vaginal smear, within 7 days		975	0.2%	508	1.1%	0.11	224	1.0%	224	1.0%	0.00
GC culture and smear, within 7 days		3153	0.6%	2366	5.3%	0.28	761	3.5%	761	3.5%	0.00
Wound swab, within 7 days		22540	4.2%	778	1.7%	0.15	589	2.7%	589	2.7%	0.00
Urine culture, within 7 days		18152	3.4%	24857	55.5%	1.39	6915	32.0%	6915	32.0%	0.00
**Recent infections**											
Urinary tract infection, within 7 days		7448	1.4%	22664	50.6%	1.35	4974	23.1%	4974	23.1%	0.00
Skin or soft tissue infection, within 7 days		7516	1.4%	2035	4.5%	0.18	1530	7.1%	1530	7.1%	0.00
Other infection, within 7 days		38176	7.2%	2288	5.1%	0.09	1308	6.1%	1308	6.1%	0.00
Sexually transmitted		2146	0.4%	707	1.6%	0.12	321	1.5%	321	1.5%	0.00

Variable	Value	Pre-weighting					Post-weighting				
		amoxicillin		TMP-SMX		Stan. Diff.	amoxicillin		TMP-SMX		Stan. Diff.
		N=530,417		N=44,801			N=21,579		N=21,579		
		n	%	n	%		n	%	n	%	
infection, within 7 days											
Acne vulgaris, within 7 days		1822	0.3%	778	1.7%	0.14	286	1.3%	286	1.3%	0.00
Acne treatment, within 7 days		3691	0.7%	471	1.1%	0.04	214	1.0%	214	1.0%	0.00
Dental caries and procedures, within 7 days		3794	0.7%	23	0.1%	0.10	21	0.1%	21	0.1%	0.00
Gingivitis or periodontitis, within 7 days		1350	0.3%	6	0.0%	0.08	6	0.0%	6	0.0%	0.00
Eye infection, within 7 days		2302	0.4%	134	0.3%	0.02	110	0.5%	110	0.5%	0.00
Bone infection, within 7 days		567	0.1%	78	0.2%	0.03	44	0.2%	44	0.2%	0.00
ENT infection, within 7 days		221393	41.7%	2401	5.4%	0.95	2281	10.6%	2281	10.6%	0.00
Rosacea, within 7 days		78	0.0%	22	0.0%	0.32	10	0.0%	10	0.0%	0.00
Eczema, within 7 days		2622	0.5%	418	0.9%	0.05	288	1.3%	288	1.3%	0.00
CBC test, within 7 days		10663	2.0%	1691	3.8%	0.11	988	4.6%	988	4.6%	0.00

Variable	Value	Pre-weighting					Post-weighting				
		amoxicillin		TMP-SMX		Stan. Diff.	amoxicillin		TMP-SMX		Stan. Diff.
		N=530,417		N=44,801			N=21,579		N=21,579		
		n	%	n	%		n	%	n	%	
OHIP record, within 7 days		319250	60.2%	37500	83.7%	0.54	15741	72.9%	15741	72.9%	0.00

eTable 9. Baseline characteristics of adolescents and young adults newly prescribed TMP-SMX versus those newly prescribed cephalosporins in Ontario, Canada (2002–2023)

Variable	Value	TMP vs CEP									
		Pre-weighting					Post-weighting				
		cephalosporins		TMP-SMX		Stan. Diff.	cephalosporins		TMP-SMX		Stan. Diff.
		N=197,039		N=51,197			N=20,538		N=20,538		
		n	%	n	%		n	%	n	%	
**Demographics**											
Age	Mean ± SD	18.25	4.02	18.94	3.8	0.18	18.68	3.95	18.68	3.98	0.00
	Median (IQR)	19	(15-22)	20	(16-22)		19	(16-22)	19	(16-22)	
	10-14	42515	21.6%	7334	14.3%	0.19	3609	17.6%	3609	17.6%	0.00
	15-19	68760	34.9%	18202	35.6%	0.01	7006	34.1%	7006	34.1%	0.00
	20+	85764	43.5%	25661	50.1%	0.13	9923	48.3%	9923	48.3%	0.00
Sex	F	106156	53.9%	42267	82.6%	0.65	14847	72.3%	14847	72.3%	0.00
	M	90883	46.1%	8930	17.4%	0.65	5691	27.7%	5691	27.7%	0.00
Income quintile	Missing	738	0.4%	226	0.4%	0.00	* imputed as '1'		* imputed as '1'		
	1	56182	28.5%	15328	29.9%	0.03	6396	31.1%	6396	31.1%	0.00
	2	39966	20.3%	10496	20.5%	0.00	4252	20.7%	4252	20.7%	0.00
	3	36140	18.3%	9232	18.0%	0.01	3704	18.0%	3704	18.0%	0.00
	4	33019	16.8%	8236	16.1%	0.02	3268	15.9%	3268	15.9%	0.00
	5	30994	15.7%	7679	15.0%	0.02	2918	14.2%	2918	14.2%	0.00
Rural	Missing	582	0.3%	169	0.3%	0.00	* imputed as 'N'		* imputed as 'N'		
	N	182187	92.5%	45404	88.7%	0.13	18336	89.3%	18336	89.3%	0.00
	Y	14270	7.2%	5624	11.0%	0.13	2202	10.7%	2202	10.7%	0.00
ODB plancode	OTHER	14351	7.3%	3625	7.1%	0.01	1469	7.2%	1469	7.2%	0.00
	ODSP	25932	13.2%	7558	14.8%	0.05	3130	15.2%	3130	15.2%	0.00

Variable	Value	TMP vs CEP									
		Pre-weighting					Post-weighting				
		cephalosporins		TMP-SMX		Stan. Diff.	cephalosporins		TMP-SMX		Stan. Diff.
		N=197,039		N=51,197			N=20,538		N=20,538		
		n	%	n	%		n	%	n	%	
	OW	29908	15.2%	11293	22.1%	0.18	4222	20.6%	4222	20.6%	0.00
	OHIP+	126848	64.4%	28721	56.1%	0.17	11718	57.1%	11718	57.1%	0.00
LHIN	1	16065	8.2%	3684	7.2%	0.04	1657	8.1%	1657	8.1%	0.00
	2	12667	6.4%	4274	8.3%	0.07	1572	7.7%	1572	7.7%	0.00
	3	11856	6.0%	3229	6.3%	0.01	1275	6.2%	1275	6.2%	0.00
	4	22805	11.6%	6727	13.1%	0.05	2683	13.1%	2683	13.1%	0.00
	5	16827	8.5%	3091	6.0%	0.10	1408	6.9%	1408	6.9%	0.00
	6	15803	8.0%	2941	5.7%	0.09	1293	6.3%	1293	6.3%	0.00
	7	14469	7.3%	3105	6.1%	0.05	1333	6.5%	1333	6.5%	0.00
	8	25139	12.8%	4548	8.9%	0.13	1980	9.6%	1980	9.6%	0.00
	9	25216	12.8%	4799	9.4%	0.11	2142	10.4%	2142	10.4%	0.00
	10	6354	3.2%	2613	5.1%	0.10	939	4.6%	939	4.6%	0.00
	11	14078	7.1%	5550	10.8%	0.13	1828	8.9%	1828	8.9%	0.00
	12	6382	3.2%	2092	4.1%	0.05	790	3.8%	790	3.8%	0.00
	13	6872	3.5%	3359	6.6%	0.14	1151	5.6%	1151	5.6%	0.00
	14	2506	1.3%	1185	2.3%	0.08	486	2.4%	486	2.4%	0.00
Year of entry	2002-2007	21763	11.0%	8640	16.9%	0.17	3179	15.5%	3179	15.5%	0.00
	2008-2013	25189	12.8%	8132	15.9%	0.09	3133	15.3%	3133	15.3%	0.00
	2014-2019	96949	49.2%	23443	45.8%	0.07	8966	43.7%	8966	43.7%	0.00
	2020+	53138	27.0%	10982	21.5%	0.13	5260	25.6%	5260	25.6%	0.00

Variable	Value	TMP vs CEP									
		Pre-weighting					Post-weighting				
		cephalosporins		TMP-SMX		Stan. Diff.	cephalosporins		TMP-SMX		Stan. Diff.
		N=197,039		N=51,197			N=20,538		N=20,538		
		n	%	n	%		n	%	n	%	
Prescriber specialty	MISSING	20269	10.3%	7834	15.3%	0.15	1330	6.5%	1330	6.5%	0.00
	OTHER	11128	5.6%	2432	4.8%	0.04	74	0.4%	74	0.4%	0.00
	DERMATOLOGY	1136	0.6%	152	0.3%	0.04	15797	76.9%	15797	76.9%	0.00
	PCP	164506	83.5%	40779	79.7%	0.10	3338	16.3%	3338	16.3%	0.00
**Medication use (120 days prior to the index date)**											
Number of days supplied	3-7	127825	64.9%	40248	78.6%	0.31	14720	71.7%	14720	71.7%	0.00
	8-14	67907	34.5%	8278	16.2%	0.43	5064	24.7%	5064	24.7%	0.00
	15+	1307	0.7%	2671	5.2%	0.27	754	3.7%	754	3.7%	0.00
Benzodiazepines		3664	1.9%	1300	2.5%	0.04	508	2.5%	508	2.5%	0.00
Antipsychotics		7092	3.6%	2053	4.0%	0.02	911	4.4%	911	4.4%	0.00
Opioids		6647	3.4%	2023	4.0%	0.03	836	4.1%	836	4.1%	0.00
Selective serotonin reuptake inhibitor		13790	7.0%	5083	9.9%	0.10	1939	9.4%	1939	9.4%	0.00
Corticoid and combinations		18704	9.5%	4398	8.6%	0.03	1904	9.3%	1904	9.3%	0.00
Estrogen and combinations		12699	6.4%	8260	16.1%	0.31	2254	11.0%	2254	11.0%	0.00

Variable	Value	TMP vs CEP									
		Pre-weighting					Post-weighting				
		cephalosporins		TMP-SMX		Stan. Diff.	cephalosporins		TMP-SMX		Stan. Diff.
		N=197,039		N=51,197			N=20,538		N=20,538		
		n	%	n	%		n	%	n	%	
Proton pump inhibitor		3793	1.9%	1316	2.6%	0.05	552	2.7%	552	2.7%	0.00
Histamine H2 receptor antagonist		1414	0.7%	571	1.1%	0.04	231	1.1%	231	1.1%	0.00
Beta agonist and combinations		7599	3.9%	2094	4.1%	0.01	891	4.3%	891	4.3%	0.00
Immunosuppressive agents		7830	4.0%	2427	4.7%	0.03	960	4.7%	960	4.7%	0.00
Antifungal agent		1133	0.6%	727	1.4%	0.08	217	1.1%	217	1.1%	0.00
Other antibiotics		4047	2.1%	1836	3.6%	0.09	638	3.1%	638	3.1%	0.00
Number of unique drug names	Mean ± SD	1	1.57	1.38	1.75	0.23	1.29	1.79	1.29	1.77	0.00
	Median (IQR)	0	(0-1)	1	(0-2)		1	(0-2)	1	(0-2)	
	0	107516	54.6%	20527	40.1%	0.29	9242	45.0%	9242	45.0%	0.00
	1-2	63284	32.1%	20847	40.7%	0.18	7597	37.0%	7597	37.0%	0.00
	3-4	18413	9.3%	6809	13.3%	0.13	2516	12.2%	2516	12.2%	0.00
	5+	7826	4.0%	3014	5.9%	0.09	1183	5.8%	1183	5.8%	0.00

Variable	Value	TMP vs CEP									
		Pre-weighting					Post-weighting				
		cephalosporins		TMP-SMX		Stan. Diff.	cephalosporins		TMP-SMX		Stan. Diff.
		N=197,039		N=51,197			N=20,538		N=20,538		
		n	%	n	%		n	%	n	%	
**Comorbidities (5 years prior to the index date)**											
Obesity		10208	5.2%	2195	4.3%	0.04	989	4.8%	989	4.8%	0.00
DM		2167	1.1%	457	0.9%	0.02	216	1.1%	216	1.1%	0.00
Depression		22236	11.3%	7958	15.5%	0.12	3040	14.8%	3040	14.8%	0.00
Hypothyroidism		3654	1.9%	1015	2.0%	0.01	411	2.0%	411	2.0%	0.00
Alcohol misuse		3267	1.7%	1093	2.1%	0.03	450	2.2%	450	2.2%	0.00
Cancer		1469	0.7%	721	1.4%	0.07	223	1.1%	223	1.1%	0.00
Schizophrenia		4837	2.5%	1403	2.7%	0.01	625	3.0%	625	3.0%	0.00
Bipolar disorder		7544	3.8%	2589	5.1%	0.06	1045	5.1%	1045	5.1%	0.00
Urinary tract infection		40258	20.4%	26291	51.4%	0.68	7780	37.9%	7780	37.9%	0.00
Skin or soft tissue infection		47738	24.2%	9890	19.3%	0.12	4433	21.6%	4433	21.6%	0.00
Other infection		96903	49.2%	29725	58.1%	0.18	11488	55.9%	11488	55.9%	0.00
Acne vulgaris		34463	17.5%	9109	17.8%	0.01	3487	17.0%	3487	17.0%	0.00
Acne treatment		5577	2.8%	1389	2.7%	0.01	552	2.7%	552	2.7%	0.00

Variable	Value	TMP vs CEP									
		Pre-weighting					Post-weighting				
		cephalosporins		TMP-SMX		Stan. Diff.	cephalosporins		TMP-SMX		Stan. Diff.
		N=197,039		N=51,197			N=20,538		N=20,538		
		n	%	n	%		n	%	n	%	
Sexually transmitted infection		38119	19.3%	13097	25.6%	0.15	4738	23.1%	4738	23.1%	0.00
Infective endocarditis		128	0.1%	30	0.1%	0.00	14	0.1%	14	0.1%	0.00
Dental caries and procedures		12200	6.2%	3704	7.2%	0.04	1487	7.2%	1487	7.2%	0.00
Gingivitis or periodontitis		1443	0.7%	395	0.8%	0.01	169	0.8%	169	0.8%	0.00
Eye infection		29506	15.0%	7311	14.3%	0.02	2917	14.2%	2917	14.2%	0.00
Bone infection		23118	11.7%	6123	12.0%	0.01	2483	12.1%	2483	12.1%	0.00
ENT infection		138311	70.2%	36846	72.0%	0.04	14563	70.9%	14563	70.9%	0.00
Rosacea		3638	1.8%	1008	2.0%	0.01	407	2.0%	407	2.0%	0.00
Eczema		62859	31.9%	15781	30.8%	0.02	6342	30.9%	6342	30.9%	0.00
Major surgery		2161	1.1%	673	1.3%	0.02	266	1.3%	266	1.3%	0.00
Fracture		8765	4.4%	1760	3.4%	0.05	786	3.8%	786	3.8%	0.00
Charlson Comorbidity Index	Mean ± SD	0.01	0.17	0.04	0.33	0.11	0.03	0.27	0.03	0.27	0.00
	Median (IQR)	0	(0-0)	0	(0-0)		0	(0-0)	0	(0-0)	
	0	195427	99.2%	50247	98.1%	0.10	20247	98.6%	20247	98.6%	0.00

Variable	Value	TMP vs CEP									
		Pre-weighting					Post-weighting				
		cephalosporins N=197,039		TMP-SMX N=51,197		Stan. Diff.	cephalosporins N=20,538		TMP-SMX N=20,538		Stan. Diff.
		n	%	n	%		n	%	n	%	
	1	1102	0.6%	337	0.7%	0.01	137	0.7%	137	0.7%	0.00
	2	377	0.2%	458	0.9%	0.09	113	0.5%	113	0.5%	0.00
	3+	133	0.1%	155	0.3%	0.04	41	0.2%	41	0.2%	0.00
**Healthcare visits/tests (365 days prior to the index date)**											
Primary care provider visits	Mean ± SD	4.62	5.98	5.91	7.45	0.19	5.69	7.54	5.69	7.7	0.00
	Median (IQR)	3	(1-6)	4	(2-8)		4	(2-7)	4	(2-7)	
	0	26618	13.5%	4516	8.8%	0.15	2210	10.8%	2210	10.8%	0.00
	1	29442	14.9%	5751	11.2%	0.11	2502	12.2%	2502	12.2%	0.00
	2	27255	13.8%	6099	11.9%	0.06	2497	12.2%	2497	12.2%	0.00
	3+	113724	57.7%	34831	68.0%	0.21	13329	64.9%	13329	64.9%	0.00
Hospitalizations	Mean ± SD	0.07	0.37	0.11	0.52	0.09	0.11	0.48	0.11	0.48	0.00
	Median (IQR)	0	(0-0)	0	(0-0)		0	(0-0)	0	(0-0)	
	0	185524	94.2%	47079	92.0%	0.09	18860	91.8%	18860	91.8%	0.00
	1	9548	4.8%	3222	6.3%	0.07	1344	6.5%	1344	6.5%	0.00
	2	1379	0.7%	536	1.0%	0.03	221	1.1%	221	1.1%	0.00
	3+	588	0.3%	360	0.7%	0.06	113	0.5%	113	0.5%	0.00
ED visits	Mean ± SD	0.64	1.47	0.91	1.8	0.16	0.91	1.85	0.91	1.82	0.00
	Median (IQR)	0	(0-1)	0	(0-1)		0	(0-1)	0	(0-1)	
	0	134260	68.1%	30626	59.8%	0.17	12354	60.2%	12354	60.2%	0.00

Variable	Value	TMP vs CEP									
		Pre-weighting					Post-weighting				
		cephalosporins		TMP-SMX		Stan. Diff.	cephalosporins		TMP-SMX		Stan. Diff.
		N=197,039		N=51,197			N=20,538		N=20,538		
		n	%	n	%		n	%	n	%	
	1	35768	18.2%	10445	20.4%	0.06	4098	20.0%	4098	20.0%	0.00
	2	13530	6.9%	4568	8.9%	0.07	1836	8.9%	1836	8.9%	0.00
	3+	13481	6.8%	5558	10.9%	0.14	2250	11.0%	2250	11.0%	0.00
Dermatologist visits	Mean ± SD	0.11	0.74	0.08	0.55	0.05	0.08	0.57	0.08	0.61	0.00
	Median (IQR)	0	(0-0)	0	(0-0)		0	(0-0)	0	(0-0)	
	0	186979	94.9%	49075	95.9%	0.05	19639	95.6%	19639	95.6%	0.00
	1	6052	3.1%	1339	2.6%	0.03	568	2.8%	568	2.8%	0.00
	2	1776	0.9%	379	0.7%	0.02	159	0.8%	159	0.8%	0.00
	3+	2232	1.1%	404	0.8%	0.03	173	0.8%	173	0.8%	0.00
Internist visits	Mean ± SD	0.11	0.72	0.16	1.19	0.05	0.15	0.92	0.15	0.93	0.00
	Median (IQR)	0	(0-0)	0	(0-0)		0	(0-0)	0	(0-0)	
	0	185352	94.1%	47278	92.3%	0.07	18961	92.3%	18961	92.3%	0.00
	1	7913	4.0%	2493	4.9%	0.04	1014	4.9%	1014	4.9%	0.00
	2	1992	1.0%	716	1.4%	0.04	287	1.4%	287	1.4%	0.00
	3+	1782	0.9%	710	1.4%	0.05	277	1.3%	277	1.3%	0.00
CT abdomen		1977	1.0%	919	1.8%	0.07	351	1.7%	351	1.7%	0.00
CT head		3818	1.9%	1275	2.5%	0.04	502	2.4%	502	2.4%	0.00
CT pelvis		2009	1.0%	913	1.8%	0.07	354	1.7%	354	1.7%	0.00
Echocardiography		3484	1.8%	1161	2.3%	0.04	444	2.2%	444	2.2%	0.00

Variable	Value	TMP vs CEP									
		Pre-weighting					Post-weighting				
		cephalosporins		TMP-SMX		Stan. Diff.	cephalosporins		TMP-SMX		Stan. Diff.
		N=197,039		N=51,197			N=20,538		N=20,538		
		n	%	n	%		n	%	n	%	
Holter monitoring		1888	1.0%	676	1.3%	0.03	249	1.2%	249	1.2%	0.00
Cervical cancer screening		12304	6.2%	7457	14.6%	0.28	2384	11.6%	2384	11.6%	0.00
Influenza vaccination		13361	6.8%	3299	6.4%	0.02	1355	6.6%	1355	6.6%	0.00
Hearing test		2326	1.2%	573	1.1%	0.01	270	1.3%	270	1.3%	0.00
TSH test		42374	21.5%	12596	24.6%	0.07	5091	24.8%	5091	24.8%	0.00
Calcium test		4734	2.4%	1655	3.2%	0.05	661	3.2%	661	3.2%	0.00
Cholesterol test		20728	10.5%	4933	9.6%	0.03	2122	10.3%	2122	10.3%	0.00
CBC test		56513	28.7%	17133	33.5%	0.10	6936	33.8%	6936	33.8%	0.00
**Recent tests**											
Throat swab, within 7 days		1141	0.6%	255	0.5%	0.01	136	0.7%	136	0.7%	0.00
Vaginal smear, within 7 days		613	0.3%	611	1.2%	0.10	183	0.9%	183	0.9%	0.00
GC culture and smear, within 7 days		1863	0.9%	2734	5.3%	0.26	674	3.3%	674	3.3%	0.00

Variable	Value	TMP vs CEP									
		Pre-weighting					Post-weighting				
		cephalosporins		TMP-SMX		Stan. Diff.	cephalosporins		TMP-SMX		Stan. Diff.
		N=197,039		N=51,197			N=20,538		N=20,538		
		n	%	n	%		n	%	n	%	
Wound swab, within 7 days		6210	3.2%	813	1.6%	0.10	595	2.9%	595	2.9%	0.00
Urine culture, within 7 days		9854	5.0%	28876	56.4%	1.34	5543	27.0%	5543	27.0%	0.00
**Recent infections**											
Urinary tract infection, within 7 days		5317	2.7%	26393	51.6%	1.32	4133	20.1%	4133	20.1%	0.00
Skin or soft tissue infection, within 7 days		56351	28.6%	2057	4.0%	0.71	1956	9.5%	1956	9.5%	0.00
Other infection, within 7 days		5574	2.8%	2606	5.1%	0.12	1205	5.9%	1205	5.9%	0.00
Sexually transmitted infection, within 7 days		1914	1.0%	807	1.6%	0.05	358	1.7%	358	1.7%	0.00
Acne vulgaris, within 7 days		4170	2.1%	856	1.7%	0.03	430	2.1%	430	2.1%	0.00

Variable	Value	TMP vs CEP									
		Pre-weighting					Post-weighting				
		cephalosporins		TMP-SMX		Stan. Diff.	cephalosporins		TMP-SMX		Stan. Diff.
		N=197,039		N=51,197			N=20,538		N=20,538		
		n	%	n	%		n	%	n	%	
Acne treatment, within 7 days		2483	1.3%	539	1.1%	0.02	243	1.2%	243	1.2%	0.00
Dental caries and procedures, within 7 days		358	0.2%	27	0.1%	0.03	19	0.1%	19	0.1%	0.00
Gingivitis or periodontitis, within 7 days		133	0.1%	8	0.0%	0.04	7	0.0%	7	0.0%	0.00
Eye infection, within 7 days		4436	2.3%	150	0.3%	0.18	132	0.6%	132	0.6%	0.00
Bone infection, within 7 days		789	0.4%	77	0.2%	0.04	52	0.3%	52	0.3%	0.00
ENT infection, within 7 days		35119	17.8%	2737	5.3%	0.40	2286	11.1%	2286	11.1%	0.00
Rosacea, within 7 days		251	0.1%	20	0.0%	0.04	15	0.1%	15	0.1%	0.00
Eczema, within 7 days		8677	4.4%	463	0.9%	0.22	385	1.9%	385	1.9%	0.00
CBC test, within 7 days		5506	2.8%	1935	3.8%	0.06	877	4.3%	877	4.3%	0.00

Variable	Value	TMP vs CEP									
		Pre-weighting					Post-weighting				
		cephalosporins		TMP-SMX		Stan. Diff.	cephalosporins		TMP-SMX		Stan. Diff.
		N=197,039		N=51,197			N=20,538		N=20,538		
		n	%	n	%		n	%	n	%	
OHIP record, within 7 days		173388	88.0%	42924	83.8%	0.12	15926	77.5%	15926	77.5%	0.00

eTable 10. Risk of secondary outcomes (hospital visit with acute respiratory failure diagnosis, mechanical ventilation, tracheotomy, or ECMO) in adolescents and young adults within 30 days of starting a new prescription for TMP-SMX vs amoxicillin and cephalosporins

	**Unweighted**		**Weighted[a]**			
	**No. events (%)**		**No. events (%)**			
**Secondary outcomes**	**TMP-SMX (n = 44801)**	**Amoxicillin (n = 530417)**	**TMP-SMX (n = 21579)**	**Amoxicillin (n = 21579)**	**Risk difference, % (95% CI)**	**Risk ratio (95% CI)**
Hospital visit with acute respiratory failure diagnosis	<6 (<0.01)	9 (0.22)	0 (0.00)	1 (0.00)	-0.004 (-0.01 to 0.002)	0.23 (0.02 to 2.26)
Hospital visit with mechanical ventilation, ECMO, or tracheotomy	13 (0.03)	49 (0.01)	7 (0.03)	2 (0.01)	0.02 (-0.001 to 0.04)	2.70 (0.96 to 7.56)
All-cause hospitalization	759 (1.69)	4386 (0.83)	374 (1.73)	522 (2.42)	-0.69 (-0.89 to -0.49)	0.72 (0.65 to 0.79)
All-cause mortality	<6 (<0.01)	16 (0.00)	3 (0.01)	2 (0.01)	0.005 (-0.01 to 0.02)	1.63 (0.40 to 6.71)
	**Unweighted**		**Weighted[a]**			
	**No. events (%)**		**No. events (%)**			
	**TMP-SMX (n = 51197)**	**Cephalosporins (n = 197039)**	**TMP-SMX (n = 20538)**	**Cephalosporins (n = 20538)**	**Risk difference, % (95% CI)**	**Risk ratio (95% CI)**

Hospital visit with acute respiratory failure diagnosis	<6 (<0.01)	7 (0.00)	1 (0.01)	2 (0.01)	-0.001 (-0.01 to 0.01)	0.87 (0.13 to 5.80)
Hospital visit with mechanical ventilation, ECMO, or tracheotomy	15 (0.03)	19 (0.01)	7 (0.04)	2 (0.01)	0.03 (0.008 to 0.05)	4.01 (1.72 to 9.30)
All-cause hospitalization	847 (1.65)	2535 (1.29)	361 (1.76)	486 (2.36)	-0.61 (-0.81 to -0.41)	0.74 (0.67 to 0.82)
All-cause mortality	<6 (<0.01)	<6 (0.00)	2 (0.01)	1 (0.01)	0.01 (-0.01 to 0.02)	2.19 (0.41 to 11.78)

Abbreviation: TMP-SMX, trimethoprim-sulfamethoxazole; CI, confidence interval; ECMO, extracorporeal membrane oxygenation.
[a] Overlap weighting was used to balance comparison groups on indicators of baseline health. The propensity score was estimated using multivariable logistic regression with 84 covariates chosen a priori (defined **in eTable 6 in the Supplement**). Overlap weighting allocates weights to patients in proportion to the likelihood that they belong to the opposite treatment group. Treated patients are weighted by the probability of not being treated (1 − PS), while untreated patients are weighted by the probability of being treated (PS).[12] Weighted risk ratios and 95% confidence intervals (CIs) were obtained through log-binomial regression, and weighted risk differences and 95% CIs were obtained using binomial regression with an identity link function.

eTable 11. Risk of a hospital visit with receipt of a nuclear medicine procedure (negative control) in adolescents and young adults within 30 days of starting a new prescription for TMP-SMX vs amoxicillin and cephalosporins

	Unweighted		Weighted[a]			
	No. events (%)		No. events (%)			
	TMP-SMX (n = 44801)	Amoxicillin (n = 530417)	TMP-SMX (n = 21579)	Amoxicillin (n = 21579)	Risk difference, % (95% CI)	Risk ratio (95% CI)
Hospital visit with receipt of a nuclear medicine procedure (negative control)	18 (0.04)	53 (0.01)	4 (0.02)	3 (0.02)	0.005 (-0.01 to 0.02)	1.29 (0.57 to 2.93)

	Unweighted		Weighted[a]			
	No. events (%)		No. events (%)			
	TMP-SMX (n = 51197)	Cephalosporins (n = 197039)	TMP-SMX (n = 20538)	Cephalosporins (n = 20538)	Risk difference, % (95% CI)	Risk ratio (95% CI)
Hospital visit with receipt of a nuclear medicine procedure (negative control)	17 (0.03)	47 (0.02)	4 (0.02)	8 (0.04)	-0.02 (-0.04 to 0.001)	0.49 (0.23 to 1.06)

Abbreviation: TMP-SMX, trimethoprim-sulfamethoxazole; CI, confidence interval; ECMO, extracorporeal membrane oxygenation.

[a] Overlap weighting was used to balance comparison groups on indicators of baseline health. The propensity score was estimated using multivariable logistic regression with 84 covariates chosen a priori (defined **in eTable 6 in the Supplement**). Overlap weighting allocates weights to patients in

proportion to the likelihood that they belong to the opposite treatment group. Treated patients are weighted by the probability of not being treated (1 − PS), while untreated patients are weighted by the probability of being treated (PS).[12] Weighted risk ratios and 95% confidence intervals (CIs) were obtained through log-binomial regression, and weighted risk differences and 95% CIs were obtained using binomial regression with an identity link function.

eTable 12. Results from case-crossover analysis

		Control period		
		**No TMP-SMX**	**TMP-SMX**	**Odds Ratio (95% CI)**
**Case period**	**No TMP-SMX**	11765	101	1.45 (1.12 to 1.86)
	**TMP-SMX**	146	41	

eFigure 1. Study design diagram comparing the use of TMP-SMX versus amoxicillin or cephalosporins and the risk of acute respiratory failure in individuals aged 10 to <25

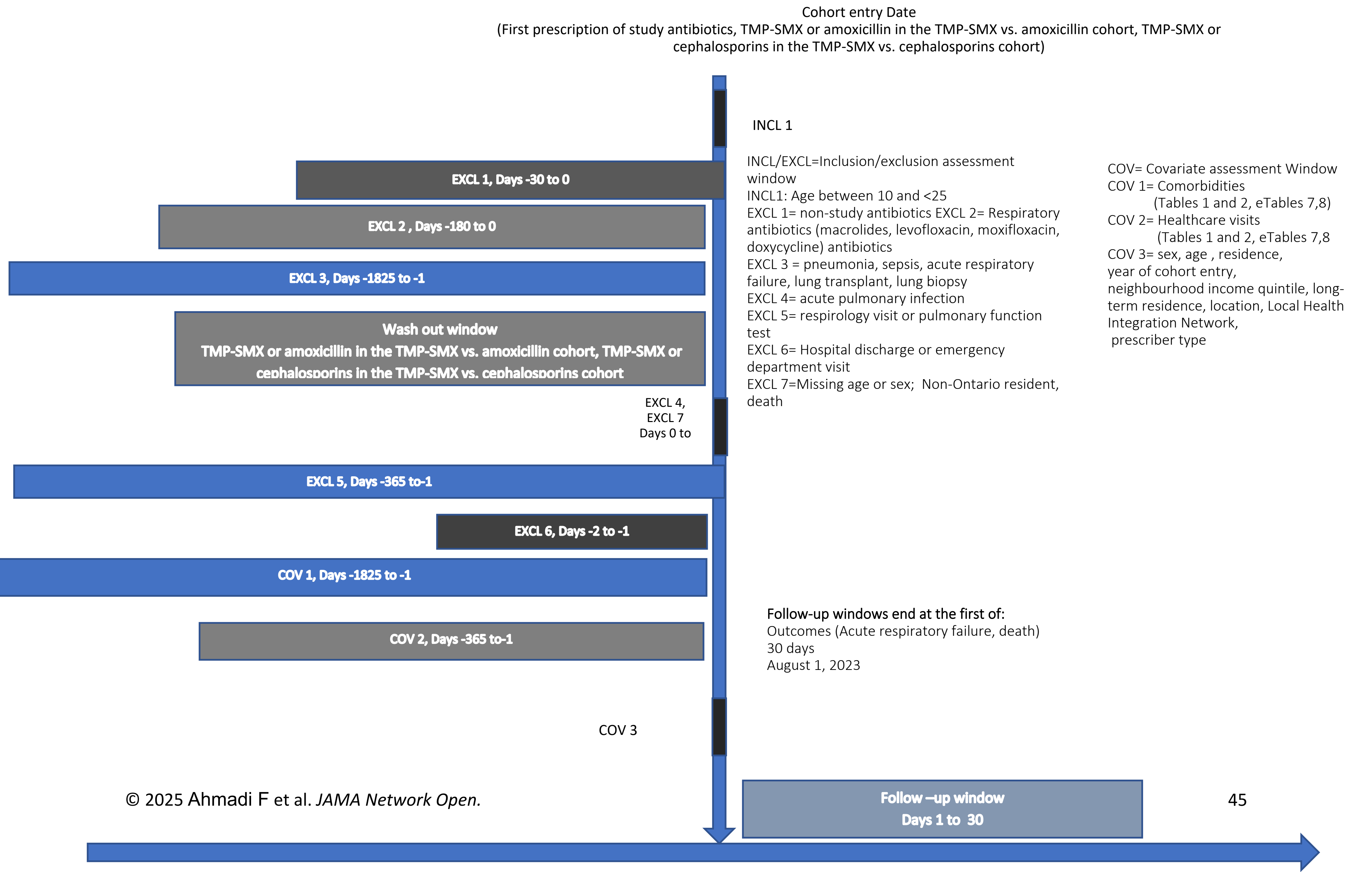

eFigure 2. Cohort build for the case-crossover study

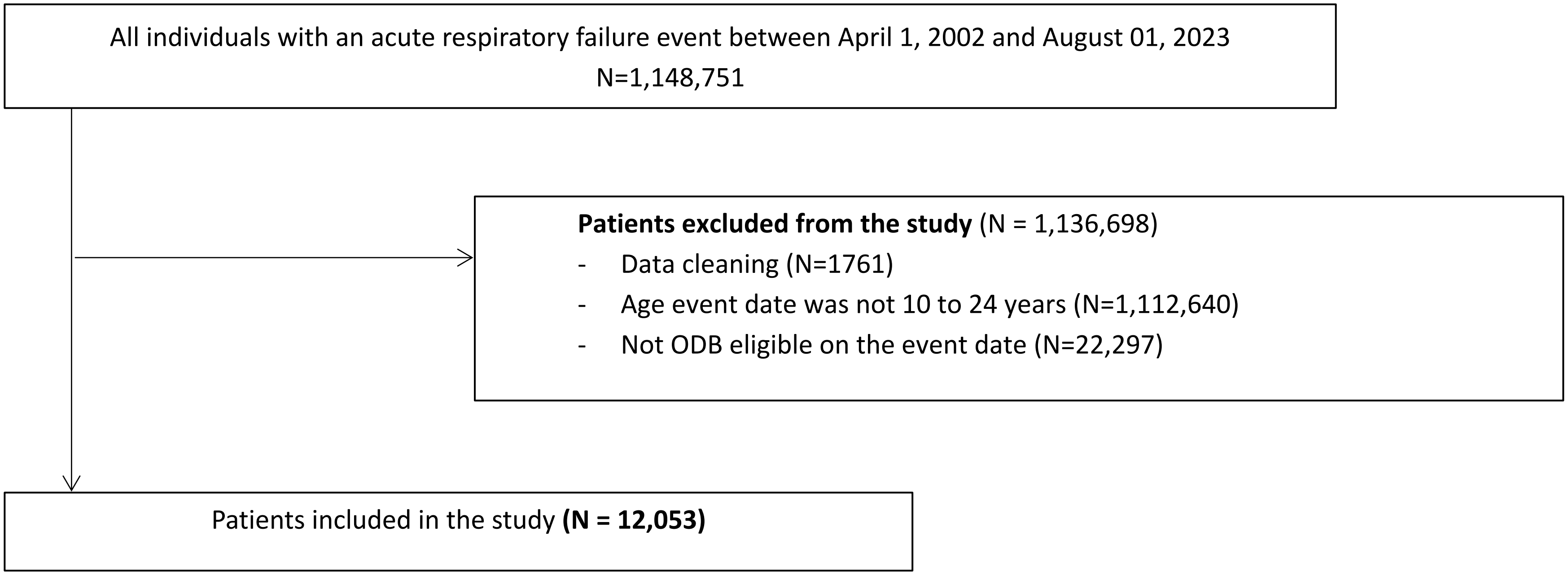

eReferences.

1. Natterer J, Rizzati F, Perez MH, Longchamp D, Amiet V, DeHalleux Q, et al. Suspected Case of Drug-Induced Acute Respiratory Distress Syndrome following Trimethoprim-Sulfamethoxazole Treatment. J Pediatr Intensive Care. 2022;11(1):67–71.

2. Ocasio V., Kapoor S., Atteih S., Sadreameli S.C., Mogayzel P.J. A Case of Trimethoprim-sulfamethoxazole Associated Fulminant Respiratory Failure in a 15- year-old Male. Am J Respir Crit Care Med [Internet]. 2024;209((Ocasio, Kapoor, Atteih, Sadreameli, Mogayzel) Eudowood Division of Pediatric Respiratory Sciences, Johns Hopkins, Baltimore, MD, United States). Available from: https://www.atsjournals.org/doi/abs/10.1164/ajrccm-conference.2024.209.1_MeetingAbstracts.A2329

3. Donnan M, Siemienowicz M, Tay HS, McLean C, Philpot S, Mason C, et al. Trimethoprim-sulfamethoxazole acute respiratory distress syndrome requiring lung transplantation. Respirol Case Rep. 2024;12(7):e01434.

4. Khanal B., Bartosek N., Calvo Ayala E. A Rare Cause of Acute Respiratory Distress Syndrome (ARDS) in Young Patients. Am J Respir Crit Care Med [Internet]. 2024;209((Khanal, Calvo Ayala) Pulmonary Disease and Critical Care Medicine, Corewell Health East William Beaumont University Hospital, Royal Oak, MI, United States(Bartosek) Internal Medicine, Corewell Health East William Beaumont University Hospital, Royal Oak,). Available from: https://www.atsjournals.org/doi/pdf/10.1164/ajrccm-conference.2024.209.1_MeetingAbstracts.A1141

5. Miller JO, Taylor J, Goldman JL. Severe Acute Respiratory Failure in Healthy Adolescents Exposed to Trimethoprim-Sulfamethoxazole. Pediatrics. 2019;143(6).

6. Persaud PN, Chhabria MS, Manek G, Nathani AR. WHEN TREATING ACNE GOES WRONG: A CASE OF BACTRIM-INDUCED ARDS IN A HEALTHY YOUNG WOMAN. Chest. 2022;162(4):A966–7.

7. Muradashvili T, Medhat G, Matcharashvili R, Ahmed M, Syndey S. Suspected Trimethoprim-sulfamethoxazole Induced Lung Injury in a Healthy Adult Female. In: B48 TOXICOLOGY AND OTHER MEDICATIONS IN THE ICU: CASE REPORTS. American Thoracic Society; 2023. p. A3510–A3510.

8. Rubin J, Chiu ML, Mino-Kenudson M, Sharma A, Witkin AS, Moschovis PP, et al. ARDS With Pneumothorax in a Young Adult. Chest. 2022;161(2):e111–6.

9. Jha P, Deboer D, Sykora K, Naylor CD. Characteristics and mortality outcomes of thrombolysis trial participants and nonparticipants: a population-based comparison. J Am Coll Cardiol. 1996;27(6):1335–42.

10. Brookhart MA, Stürmer T, Glynn RJ, Rassen J, Schneeweiss S. Confounding control in healthcare database research: challenges and potential approaches. Med Care. 2010;48(6):S114–20.

11. Desai RJ, Franklin JM. Alternative approaches for confounding adjustment in observational studies using weighting based on the propensity score: a primer for practitioners. bmj. 2019;367.

12. Thomas LE, Li F, Pencina MJ. Overlap weighting: a propensity score method that mimics attributes of a randomized clinical trial. Jama. 2020;323(23):2417–8.

13. Austin PC, Stuart EA. Moving towards best practice when using inverse probability of treatment weighting (IPTW) using the propensity score to estimate causal treatment effects in observational studies. Stat Med. 2015;34(28):3661–79.

14. Fang J. Using SAS® procedures FREQ, GENMOD, logistic, and PHREG to estimate adjusted relative risks–a case study. In Citeseer; 2011. p. 345.

15. Maclure M. The case-crossover design: a method for studying transient effects on the risk of acute events. Am J Epidemiol. 1991;133(2):144–53.
