# Supplementary material for: Trimethoprim-Sulfamethoxazole and Acute Respiratory Failure in Adolescents and Young Adults
Source: JAMA Netw Open. 2025 Nov 24;8(11):e2545251. doi: 10.1001/jamanetworkopen.2025.45251 (PMC12645330; doi:10.1001/jamanetworkopen.2025.45251)
Supplement: Supplement 2. — Data Sharing Statement [file jamanetwopen-e2545251-s002.pdf]

Data Sharing Statement

Ahmadi. Trimethoprim-Sulfamethoxazole and Acute Respiratory Failure in Adolescents and Young Adults. *JAMA Netw Open*. Published November 24, 2025.
doi:10.1001/jamanetworkopen.2025.45251

Data
Data available: No
